# Forecast of total health expenditure on China’s ageing population: a system dynamics model

**DOI:** 10.1186/s12913-024-12113-6

**Published:** 2024-12-27

**Authors:** Shihua Luo, Junlai Zhang, Mark Heffernan

**Affiliations:** 1 Jiangxi University of Finance and Economics, School of Statistics and Data Science, Nanchang, China; 2 Heidelberg University, Faculty of Medicine and University Hospital, Heidelberg Institute of Global Health(HIGH), Heidelberg, Germany; 3 Vienna University of Economics and Business(WU), Department of Economics, Vienna, Austria; 4 Western Sydney University, School of Computer, Data and Mathematical Sciences, Sydney, Australia; 5 Dynamic Operations, Sydney, Australia

**Keywords:** Total health expenditure, System dynamics, Population ageing, Total population decline, Total fertility rate, Scenario analysis, Efficiency impact factors, China GDP forecast, THE/GDP

## Abstract

**Background:**

China is currently at a turning point as its total population has started to decline, and therefore faces issues related to caring for an ageing population, which will require an increase in Total Health Expenditure (THE). Therefore, the ability to forecast China’s future THE is essential.

**Methods:**

We developed two THE System Dynamics (SD) models using Stella Architect 3.4 to simulate China’s THE from 2000 to 2060. The constant prices THE SD model estimates THE under low, medium, and high Total Fertility Rate (TFR) scenarios. The current prices THE SD model serves as a robust calibration check. In addition, we developed a new total Gross Domestic Production (GDP) forecast model to estimate THE/GDP over the same period.

**Results:**

Our simulation results reveal a significant upward trend in China’s THE from 2000 to 2060. Specifically, under the low TFR scenario, THE is projected to reach approximately $33.4 trillion in 2015 constant USD by 2060. However, with the introduction of efficiency impact factors, THE is expected to fall to around $8.6 trillion by 2060. Additionally, the per capita Health Expenditure is anticipated to rise from $102 in 2000 to roughly $30,800 by 2060, though it could see a decrease to nearly $7,900 with efficiency improvements. Our GDP forecast for 2060 is nearly $87 trillion, with THE to GDP ratio expected to be about 9.7%. In our scenario analysis, as TFR increases, the growing new births and decreased ageing rate are expected to lead to a rise in THE and a decrease in per capita Health Expenditure.

**Conclusion:**

The efficiency of THE utilization needs to be improved. Increasing TFR can help alleviate population decline and ageing to some extent. Enhancing workforce productivity and sustained economic growth is needed to counteract the challenges posed by an ageing population.

**Supplementary Information:**

The online version contains supplementary material available at 10.1186/s12913-024-12113-6.

## Background

China is currently at a crossroads of negative population growth and accelerated ageing, accompanied by a yearly increase in Total Health Expenditure (THE). This not only signifies a crucial transformation in the size and structure of the population but also highlights the growing pressure on THE [1, 2].

In 2022, China’s total population was 1.41175 billion, a decrease of 850,000 from the previous year [3]; by 2023, it further decreased by 2.08 million to 1.40967 billion, marking the first and second years of negative growth since 1961, as reported by the National Bureau of Statistics of China [4]. In 2020, the age distribution of the population was as follows: 253.38 million (17.95%) were 0–14 years old, 894.38 million (63.35%) were 15–59 years old, and those 60 and over totaled 264.02 million (18.70%), including 190.64 million (13.50%) aged 65 + . Since 2010, the 0–14 age group has increased by 1.35%, the 15–59 group decreased by 6.79%, and those over 60 increased by 5.44%. These shifts mark a significant upward trend in the ageing of the population, posing challenges for balanced population development.

Moreover, China’s THE in 2000 was 458.7 billion yuan, which surged to nearly 7.7 trillion yuan by 2021. Per capita Health Expenditure increased from 362 yuan to 5,441 yuan during this 21-year period. Similarly, the proportion of the GDP to THE rose from 4.57% in 2000 to 6.72% in 2021. Notably, despite an average annual GDP growth rate of 8.66% from 2002 to 2021, the annual growth rate of THE in the period was 10.82%, outpacing that of GDP [5].

In response to the challenges detailed above and to support health policy decisions, we examine the following core issues within the context of ageing, spanning from 2000 to 2060.

### What are the trends in China’s total health expenditure and health expenditure by age and sex?

In previous studies, a system dynamics model was used to explore changes in Germany’s population structure and their feedback effects on economic indicators, finding that stabilizing the age structure requires appropriate policy interventions, such as raising the retirement age and increasing birth rates [6]. Another study analyzed the sustainability of the Dutch government’s retirement and healthcare expenditure using system dynamics models and robust decision-making methods. It revealed that declining productivity and increased life expectancy lead to rising social costs, requiring improved productivity to maintain current social security levels [7]. It was also found that individual mixing in system dynamics models can result in large distortions related to chronological ageing and age-related characteristics. To address this, a ‘continuous cohorting’ approach was proposed to ensure accuracy in modeling population dynamics [8]. Further, a system dynamics model was used to estimate the future number and living arrangements of dementia patients in Singapore, revealing that the increase in severe dementia cases could challenge family care systems, indirectly highlighting the potential impact of population ageing on healthcare resources and costs [9]. Another study developed a system dynamics model of a social care system, finding that an increasing ageing population would impose greater challenges on the system [10].

In utilizing System Dynamics (SD) for forecasting THE, the first THE SD model was developed, comprising four subsystems: GDP growth, population growth, medical security, and medical institution assets, covering historical data from 1990 to 2004 and a simulation period from 2005 to 2009 [11]. Subsequently, the second THE SD model was established, including subsystems of population, GDP, social medical insurance, and medical institution assets, with historical data from 2010 to 2015 and a forecast period from 2016 to 2020 [12]. The third THE SD model expanded further, encompassing multiple subsystems such as THE, GDP, total population, elderly population size (65 + population), drug costs, and the number of health technicians per thousand people, with simulations covering the historical period from 2001 to 2016 and the forecast period from 2017 to 2025 [13].

In utilizing other models for forecasting THE, an Autoregressive Integrated Moving Average (ARIMA) model was used to forecast THE from 2018 to 2022, showing a rapid increase in THE and highlighting the need to improve the efficiency and fairness of health fund usage [14]. Similarly, ARIMA models were applied to forecast health spending and related indicators for BRICS countries (including China) from 2018 to 2030, revealing a long-term trend of increasing per capita spending in terms of purchasing power parity (PPP) [15]. A Current Health Expenditure Projection Model was used to estimate health spending by disease and function from 2015 to 2035, with China’s Current Health Expenditure projected to grow at an average annual rate of 8.4% during this period [16]. A set of linear mixed-effects models with time-series specifications was employed to forecast domestic health spending for 195 countries, including China, from 2017 to 2050, emphasizing that improving the efficiency of health spending is key to achieving global health goals in the absence of sustained new health investments [17].

Existing research indicates that in forecasting China’s THE, most SD models primarily focus on analyzing various factors affecting THE, with less consideration given to the impact of detailed population dynamics on forecast accuracy. Combining the SD method with population forecast models can not only expand the current analytical framework but also offer new perspectives for optimizing variables and parameters within the system [13]. Apart from this suggestion above, research on forecasting and analyzing Health Expenditure by age and sex is also relatively limited, with most literature having a relatively short forecast time frame.

Building on this premise, we pioneer the use of Australia’s per capita Health Expenditure by age and sex to estimate the Health Expenditure by age and sex index for China. By integrating the simulation results of the Array Population Model, the study forecasts and analyzes the future long-term THE. This methodological fusion provides a novel analytical framework for understanding the dynamic changes in THE. It allows for a more comprehensive understanding and forecast of long-term Health Expenditure trends, significantly enhancing the accuracy and reliability of Total Health Expenditure forecasts. In addition, the traditional Solow model emphasizes capital accumulation, workforce growth, and technological progress as the three key drivers of economic growth [18]. In our study, a “per capita GDP labor productivity index” is introduced into the model to dynamically capture the effects of workforce efficiency and technological advancement on per capita GDP. This approach offers new insights for future GDP growth modeling by projecting GDP under different TFR scenarios.

In summary, we construct an index for China’s per capita Health Expenditure by age and sex, based on the Australian Health Expenditure per capita by age and sex. Utilizing the grouped population data from the Array Population Model, we calculate the Health Expenditure grouping index. On this basis, the study, considering only the impacts of population ageing, growing healthcare demands, and rising medical costs, builds two SD models: a constant prices model calculated in 2015 United States Dollars (USD) constant prices and a current prices model calculated in current Chinese yuan prices, which forecast and analyze China’s THE. Finally, we develop a new total GDP forecast model to calculate the THE/GDP ratio.

### Methodology

#### Model applicability

**Fig. 1:**
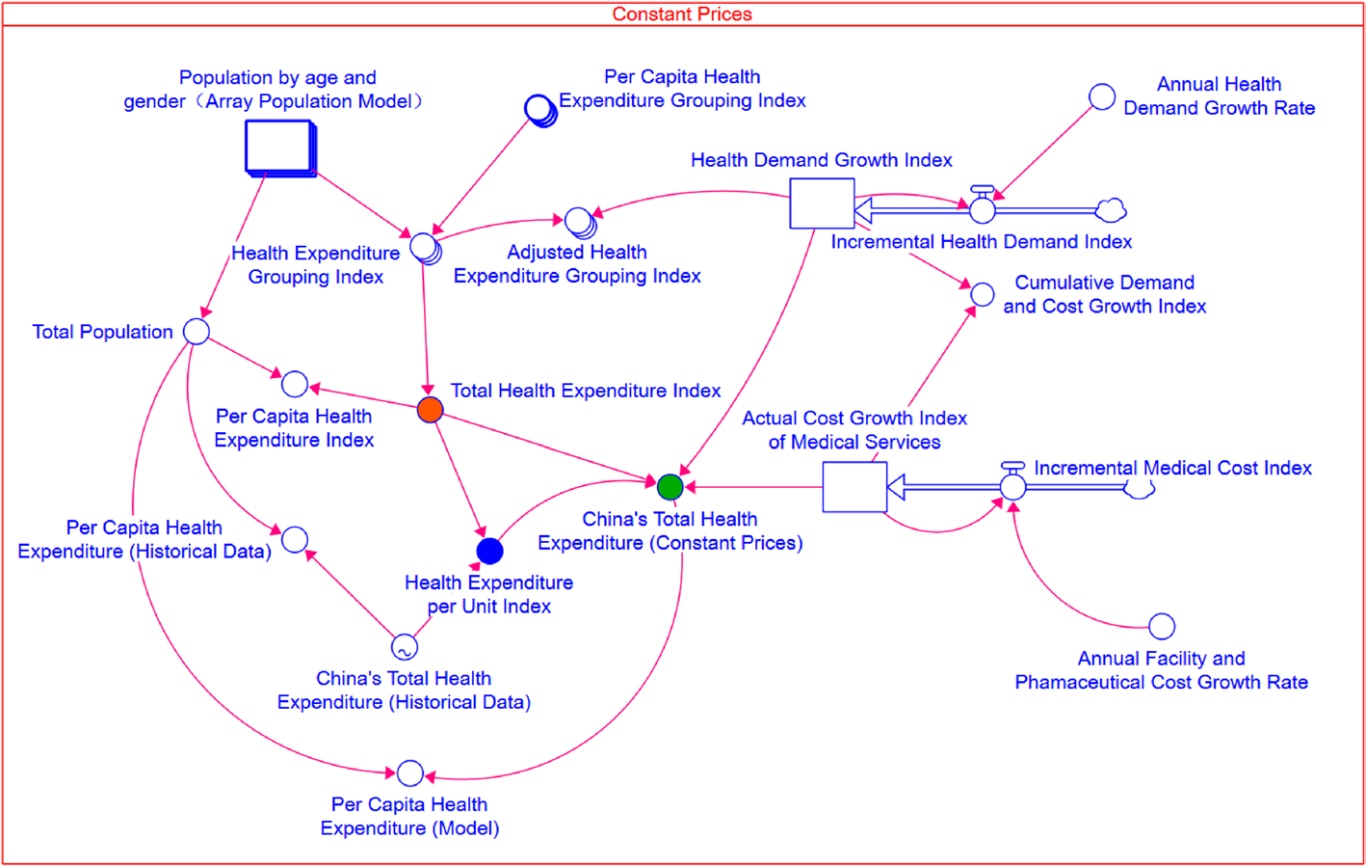
Stock-flow diagram (SFD) of the total health expenditure model (constant prices)

**Fig. 2:**
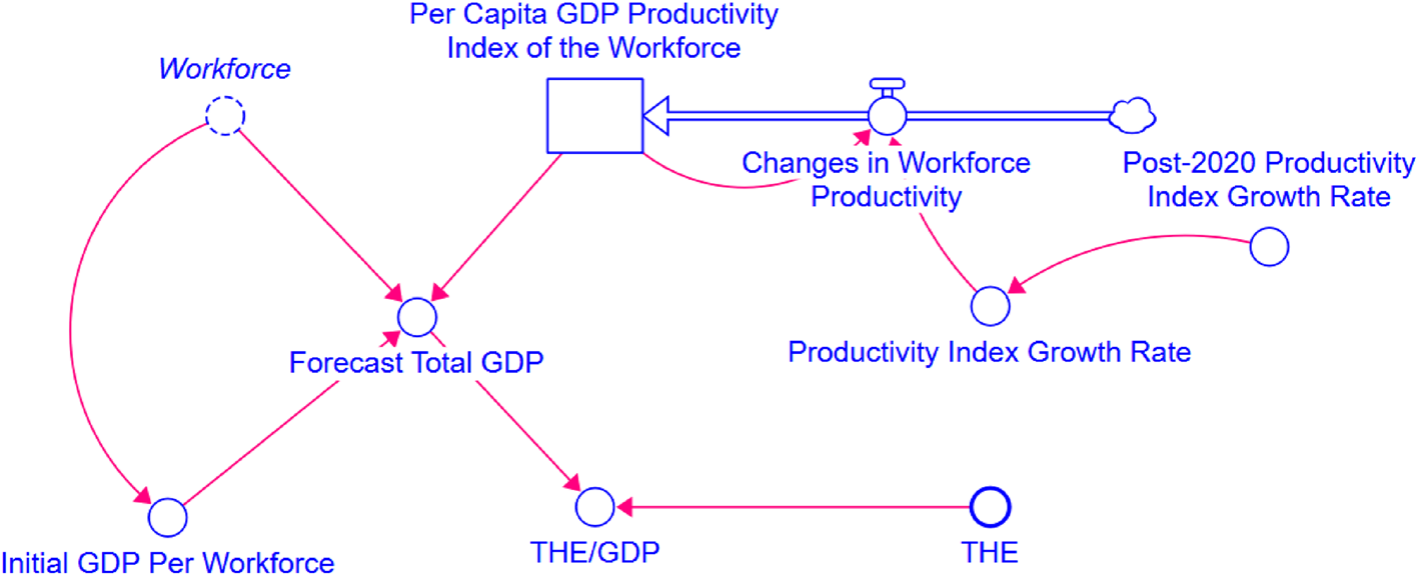
Total GDP forecasting model

System dynamics models are well-suited for capturing the dynamic feedback, nonlinear relationships, and long-term trends in complex systems. To address these complexities, we integrated an Array Population Model (Figure A6), Workforce Model (Figure A7), Total GDP Forecasting Model (Fig. 2), and Total Health Expenditure Model (Fig. 1). Additionally, our SD model can simulate long-term trends in China’s THE from 2000 to 2060. In contrast, spreadsheet models are more appropriate for simpler, short-term forecasts. System dynamics also allows for scenario analysis, which is valuable for policy-making. We forecasted population size, structure, and THE under different TFR scenarios. In comparison, spreadsheet models are less flexible in simulating multiple scenarios at the same time. System dynamics provides a refined approach to population modeling by efficiently updating 42 groups in each iteration. In contrast, a spreadsheet-based approach would require managing over 10,000 cells for a 60-year analysis, making it more prone to errors due to its inherent complexity.

#### Data sources and software

We use System Dynamics software, Stella Architect 3.4, to build two System Dynamics models at constant prices and current prices to estimate THE from 2000 to 2060.

To construct the THE models, we have utilized various data sources, including the Population by Age and Sex and Total Population in different TFR scenarios simulated by Zhang and Heffernan [19], per capita Health Expenditure by age and sex as reported by the Australian Institute of Health and Welfare [20], GDP in 2015 constant prices in USD and GDP Growth Rate as reported by the World Bank [21], Disability-adjusted Life Years by the Global Burden of Disease (GBD) [22], Percentage of THE to GDP and THE (current prices) from the China Statistical Yearbook [23]. The Annual Health Demand Growth Rate and the Annual Facility and Pharmaceutical Cost Growth Rate are from our own estimations which have been calibrated in the subsequent model calibration section. The data sources for the Total GDP Forecasting Model are as follows: working-age population data by Zhang and Heffernan [19]; workforce participation rates by Ma et al. [24]; and the initial GDP per workforce, as well as the per capita GDP productivity index are derived from our own estimates.

#### Key variables and equations

Tables A1, A2, and A3 present the main variables of the Total Health Expenditure model and Total GDP Forecasting Model, including their types, definitions, units, and equations.

### Total health expenditure system dynamics model

#### Stock-Flow diagrams

Figure 1 is a Stock-Flow Diagram (SFD) for the THE Model based on 2015 constant prices in USD. Figure A1 shows the SFD of the THE Model using current prices.

The core formula is defined as follows:

1 $$\text{Total Health Expenditure }(\text{t})=\text{Health Expenditure Per Unit Index }(\text{t}) *\text{ Total Health Expenditure Index }(\text{t}) *\text{ Health Demand Growth Index }(\text{t}) *\text{ Actual Cost Growth Index of Medical Services}(\text{t})$$

Health Expenditure Per Unit Index is defined as the Total Health Expenditure for the year 2000 divided by the Total Health Expenditure Index. Total Health Expenditure Index is calculated as the Health Expenditure per capita by Age and Sex Index multiplied by the Population by age and sex. Total Health Expenditure Index reflects population structural changes due to increased ageing, causing the overall index to rise. The Health Demand Growth Index is a stock measure that accumulates based on the Annual Health Demand Growth Rate of 3% (an auxiliary variable). Similarly, the Actual Cost Growth Index of Medical Services, also a stock measure, accumulates according to the Annual Facility and Pharmaceutical Cost Growth Rate of 5%. These indices are designed to track the compounded annual changes in health demand and medical costs, respectively.

#### Health demand growth and facility & pharmaceutical cost growth

Evidence from the literature, Baumol’s cost disease theory, and data from the China Statistical Yearbook collectively potentially forecast increases in both China’s health demand and the costs of facilities and pharmaceuticals [15, 23, 25–28]. We modeled expected growth rates of 3% for health demand and 5% for facility and pharmaceutical costs in our THE SD model at constant prices. Additionally, we modeled expected growth rates of 3% for health demand and 8% for facility and pharmaceutical costs at current prices, with the 8% accounting for inflation. These estimates are arbitrary and will require empirical calibration in future studies. Our model’s simulations of THE were calibrated using historical data and findings from related academic research, producing similar results (see Figure A2, A3, and Table A4).

#### Health expenditure per capita by age and sex index

Given the challenges in obtaining age- and sex-specific per capita Health Expenditure data for China, we developed a per capita Health Expenditure index for China as shown in Table A5, based on Australia’s age- and sex-specific data. This index, which is positively correlated with corresponding Health Expenditure, reflects the distribution of per capita Health Expenditures across different age groups. Additionally, there is a significant correlation (R^2^ = 0.9424) between the Disability-Adjusted Life Years (DALYs) rates in China and Australia (see Figure A4), indicating that the patterns of health status changes with age are similar in both countries. Consequently, the distribution of Health Expenditure across different age groups may also exhibit a certain degree of similarity between the two countries. Finally, we further calibrated our results by comparing them with historical data and estimates from other researchers, confirming their feasibility (see Figures A2, A3, and Table A4).

**THE model (constant prices):** We calculate China’s Total Health Expenditure by multiplying the GDP at 2015 constant USD from the World Bank with the THE to GDP ratio reported in the China Statistical Yearbook 2022 [21, 23]. This approach offers three advantages: 1) it allows for longitudinal analysis; 2) it enables potential international comparisons; and 3) it is a novel approach.

#### The total health expenditure as a percentage of GDP

The ratio of Total Health Expenditure to Gross Domestic Product (GDP) within the same period is a crucial indicator of a country’s health investment relative to its economic output. It reflects a nation’s intensity of investment in healthcare for a specific period and also demonstrates the level of attention given by the government and society as a whole to healthcare and public health [23]. We constructed a Total GDP Forecasting Model, as depicted in Fig.2, designed to provide an intuitive and straightforward method for forecasting GDP, enabling the subsequent calculation of the ratio of THE to GDP from 2000 to 2060.

### Total GDP forecasting model

#### Stock-Flow diagrams

Figure 2 is a Stock-Flow Diagram (SFD) for the Total GDP Forecasting Model based on 2015 constant prices in USD.

Given the decline in population size, ageing demographics, and associated economic factors, can total GDP continue to grow at its previous rates? To address this question, we have developed a Total GDP Forecasting Model, integrating workforce participation rates and a workforce productivity index with age-grouped population data from the array population model. Relevant definitions and formulas can be found in Tables A2 and A3.

The core formula is defined as follows:

2 $$\mathrm{GDP}=\mathrm{Workforce}\ast\mathrm{Initial}\;\mathrm{GDP}\;\mathrm{Per}\;\mathrm{Workforce}\ast\mathrm{Per}\;\mathrm{Capita}\;\mathrm{GDP}\;\mathrm{Productivity}\;\mathrm{Index}\;\mathrm{of}\;\mathrm{the}\;\mathrm{Workforce}$$

As the COVID-19 pandemic subsides, China’s national economy is gradually recovering and heading towards a positive rebound. Despite some fluctuations during the recovery, China’s economy is expected to achieve stable and rapid growth in the future [29]. After calibration with historical GDP data, we set the Productivity index Growth Rate at 9% for 2000–2019 and opted for a 5% rate from 2020 to 2060. The selection of these rates is preliminary and calls for further empirical research to substantiate these choices.

## Results

Consistent with previous studies [19], we follow the same classification standards for Total Fertility Rates (TFR), categorizing low, medium, and high TFR as 1.05, 1.45, and 1.85, respectively.

### Health expenditure

**Fig. 3:**
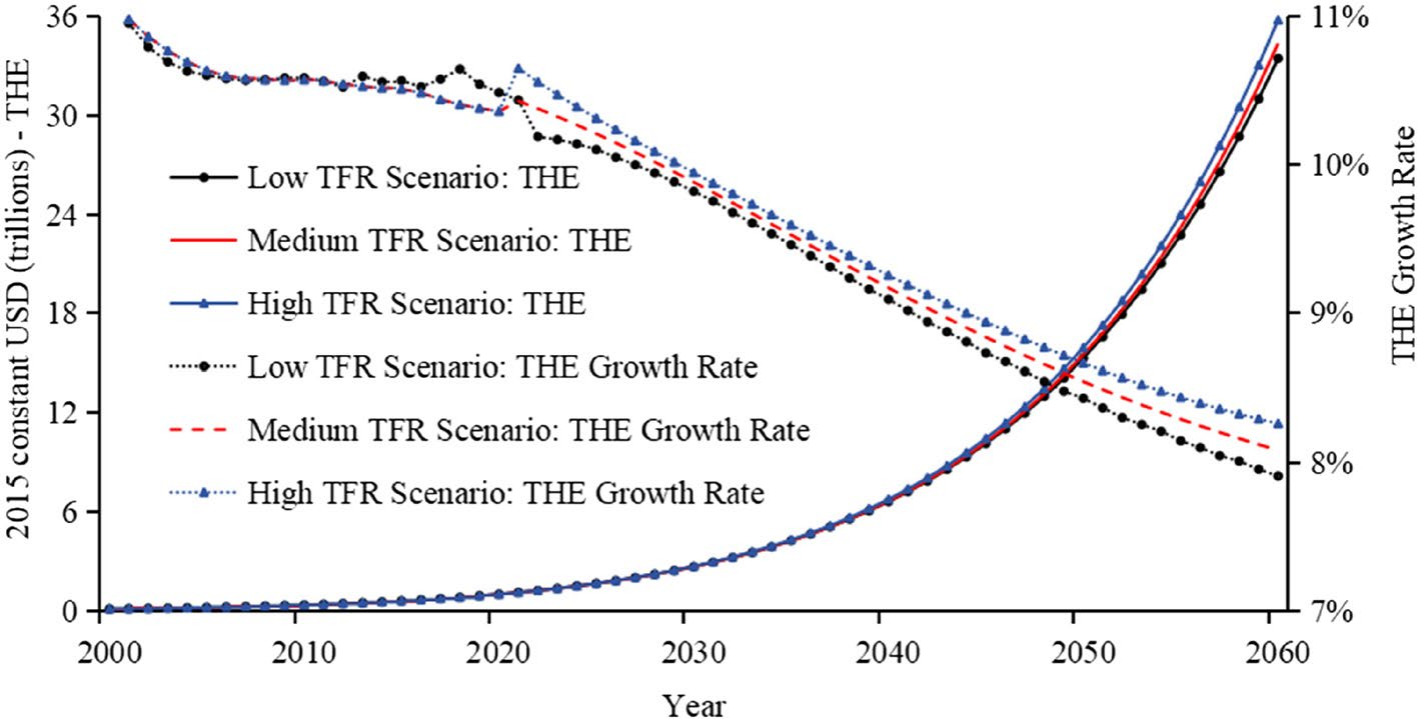
Total health expenditure and growth rate under different TFR scenarios

**Fig. 4:**
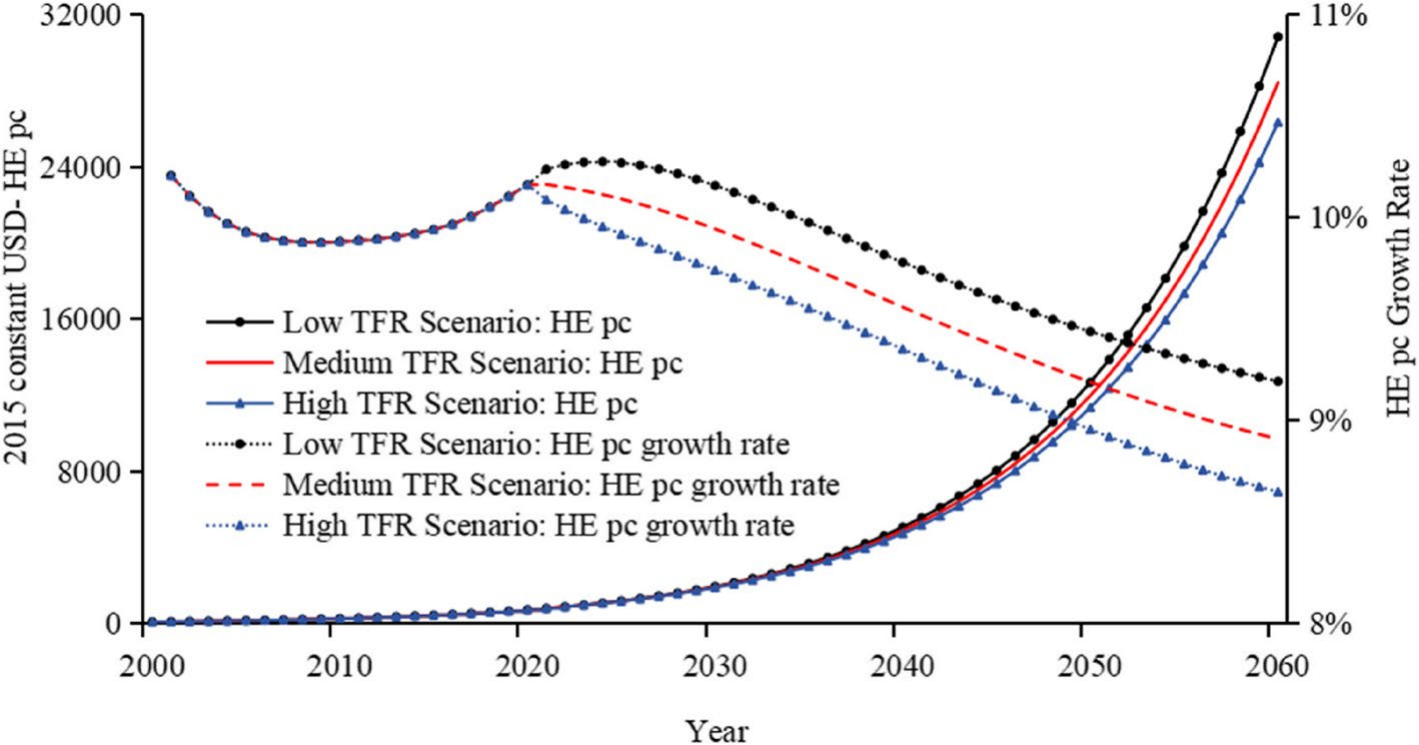
Health expenditure per capita and growth rate under different TFR scenarios

Figures 3 and 4 display the trends in THE and its growth rate, along with Health Expenditure per capita (HE pc) and its growth rate, from 2000 to 2060 under low, medium, and high TFR scenarios.

The trends in THE and its growth rate, alongside HE pc and its growth rate, are similar under low, medium, and high TFR scenarios. The low TFR scenario will be used as an example for analysis. Firstly, per capita Health Expenditure has shown a stable upward trend, rising from $102 in 2000 to roughly $30,800 by 2060. Additionally, the growth rate of per capita Health Expenditure started at 10.21% in 2001, decreased initially, then rose to a peak of nearly 10.3% in 2024, and gradually fell to nearly 9.2% by 2060, maintaining a relatively high level throughout. Secondly, the Total Population of China initially increased, rising from 1.243 billion in 2000 to 1.396 billion in 2021, then subsequently decreased to almost 1.09 billion by 2060 under a low TFR scenario. Meanwhile, the degree of population ageing intensified, as shown in Figure A11. Lastly, China’s THE for 2021 to 2060 is expected to increase significantly. By 2040, it is expected to have risen to approximately $6.6 trillion (2015 constant USD), reaching about $33.4 trillion by 2060 (2015 constant USD).

### Health expenditure grouping index under the low TFR scenario

The Health Expenditure grouping index is calculated as the product of the population in each group and the per capita Health Expenditure group index. The per capita Health Expenditure group index corresponds with the per capita Health Expenditure by age and sex. The Health Expenditure group index varies with the Health Expenditure by age and sex. This index takes into account only the factor of ageing and does not cover factors related to increasing health care expectations or real cost escalation in healthcare services.

**Fig. 5:**
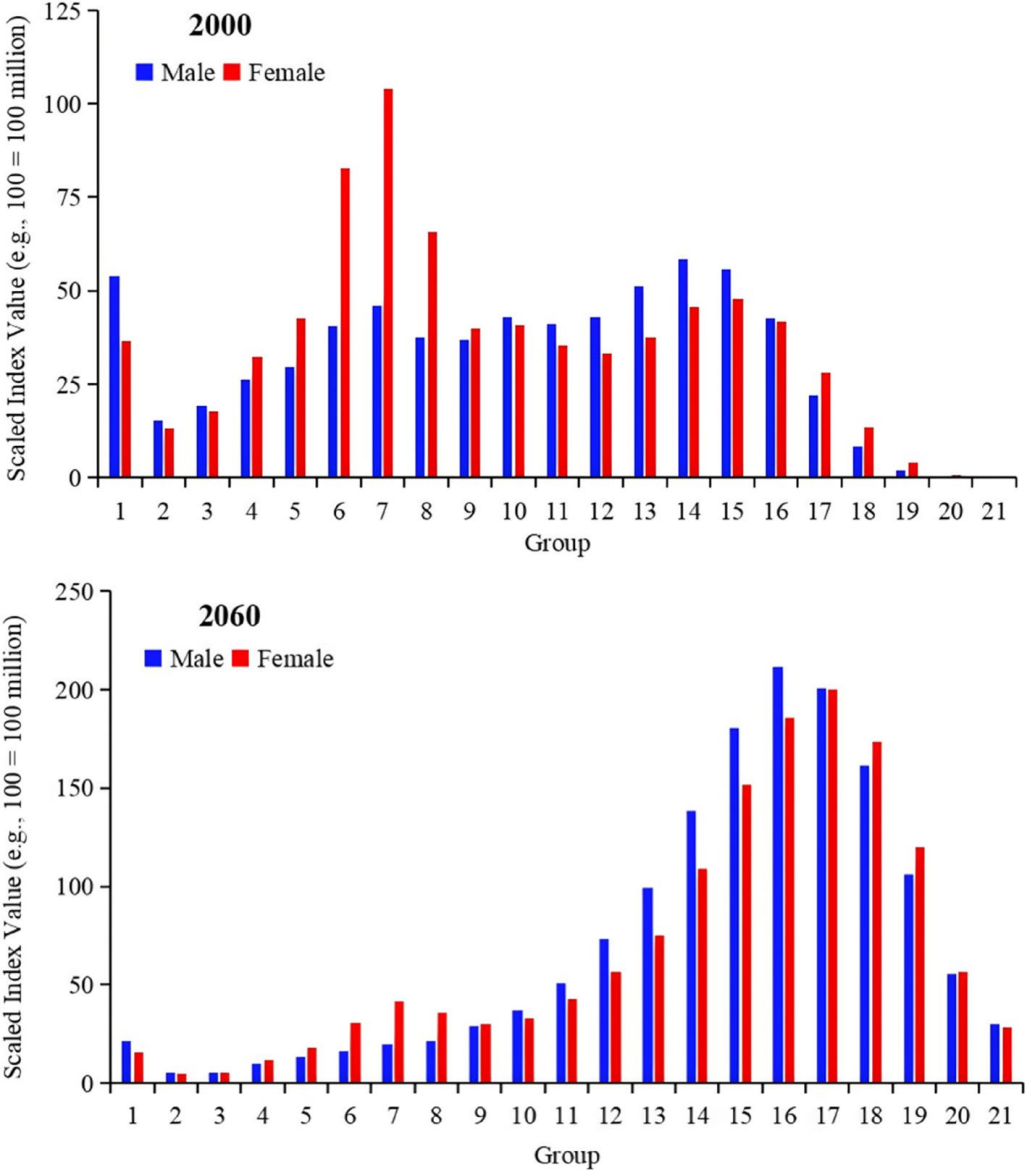
Health Expenditure Group Index under the low TFR scenario. Note: The bars are numbered from 1 to 21 (referred to as “groups”), each one representing 5 years. At the bottom left of the graph, blue and red bars, labeled as ‘1’, represent males and females aged 0 to 4.99 years in 2000 and 2060, respectively. The data presented in the figure is dimensionless, representing proportions or index values, not absolute quantities. Therefore, no units are associated with the numbers shown. The values on the y-axis are scaled down for clarity; for example, 100 represents 100 million

Regarding the Health Expenditure grouping index for the year 2000 (as illustrated in Fig. 5), the expenditure for males in the first group (ages 0–4.99) exceeds that of females in the same age group. This is attributed to the higher number of males, greater burden of disease (expressed by DALYs), and higher Health Expenditure in the first male group compared to their female counterparts. Conversely, in groups five (ages 20–24.99), six (ages 25–29.99), and seven (ages 30–34.99), expenditure for females are higher than those of males, mainly due to the higher costs associated with childbirth [30–32]. As the ageing process advances, by the year 2060 (as depicted in Fig. 4), the distribution of Health Expenditure by group exhibits a ‘right-skewed distribution’ shape. The Health Expenditure for Groups 12 (ages 55–59.99) to 19 (ages 90–94.99) is exceptionally high, with the highest Health Expenditure observed in group 16 (ages 75–79.99) for males and group 17 (ages 80–84.99) for females. By 2060, the number of women of childbearing age is expected to have significantly decreased. For instance, taking group six (ages 25–29.99), based on the low TFR scenario simulation [19], the female population in this age group is projected to drop to only 22.24 million, a significant change from 60 years before when the number of females in this age bracket was approximately 58.99 million.

A comparison of the Health Expenditure grouping index for the years 2000 and 2060 clearly illustrates the trends in Health Expenditure across different age and sex groups, as well as the intensified impact of the ageing population in China on its Health Expenditure.

### Total health expenditure index

Total Health Expenditure Index is the cumulative sum of the individual Health Expenditure grouping indices. This index primarily focuses on the impacts of population ageing and the changes in population size on THE and does not include factors such as the growth in expectation or real cost increases in health services.

**Fig. 6:**
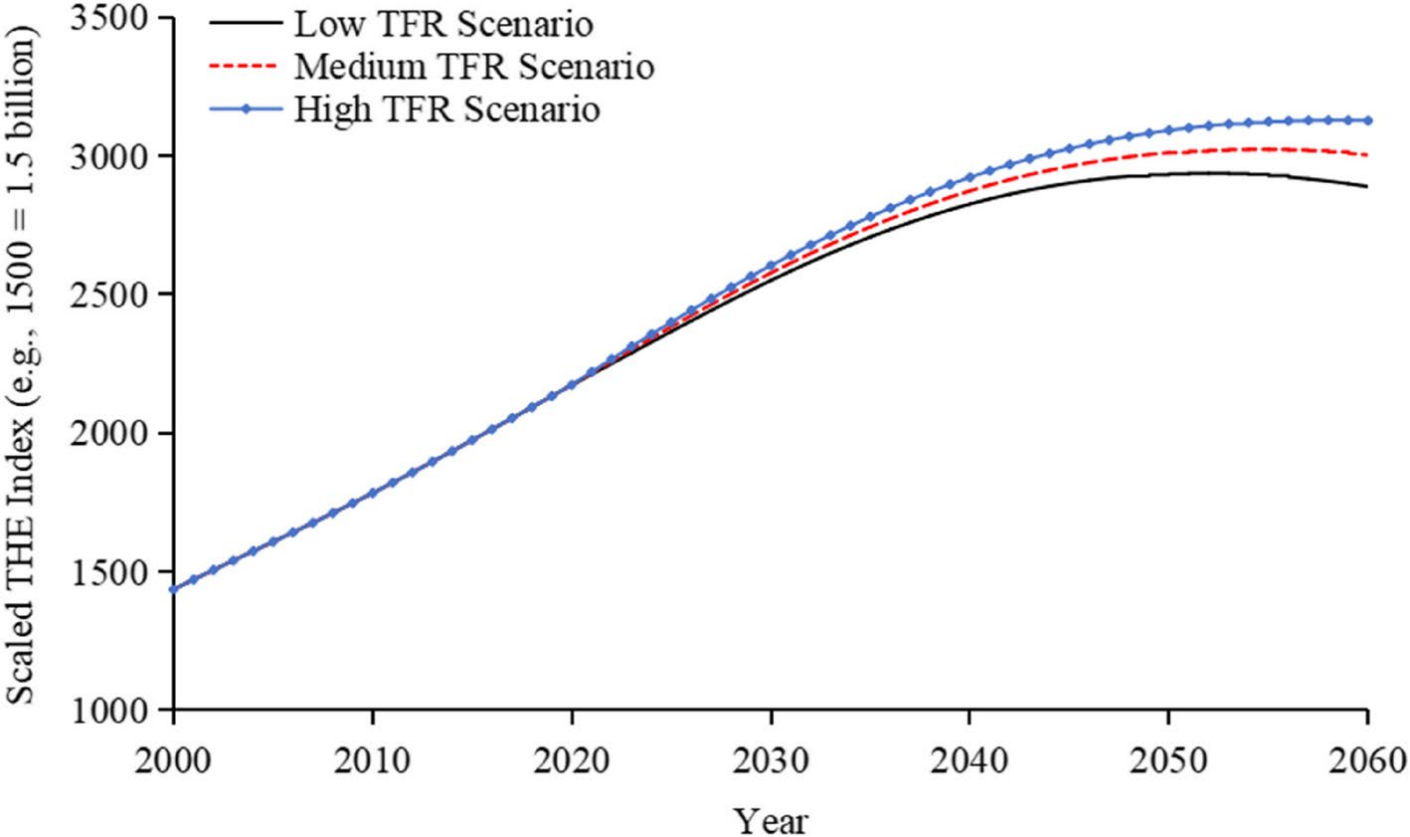
Trend of the Total Health Expenditure Index under different TFR scenarios. Note: The data presented in the figure is dimensionless, representing proportions or index values, not absolute quantities. Therefore, no units are associated with the numbers shown. The values on the y axis are scaled down for clarity; for example, 1500 represents 1.5 billion

The index shows the trends of Total Health Expenditure under low, medium, high TFR scenario as illustrated in Fig. 6. Starting from 2021, Total Health Expenditure Index begins to diverge under three different TFR scenarios. Over time, the disparity between the scenarios gradually widens, with each reaching its peak at different times. Despite these divergences, the overall trend in each scenario shows an initial increase followed by a gradual decline. Under the high TFR scenario, population size decline is gradual and population ageing progresses gradually. As a result, Total Health Expenditure Index remains relatively stable. Conversely, under the low TFR scenario, despite significant ageing, a sharp decrease in population size results in an earlier peak of Total Health Expenditure Index.

### GDP Forecast results

**Fig. 7:**
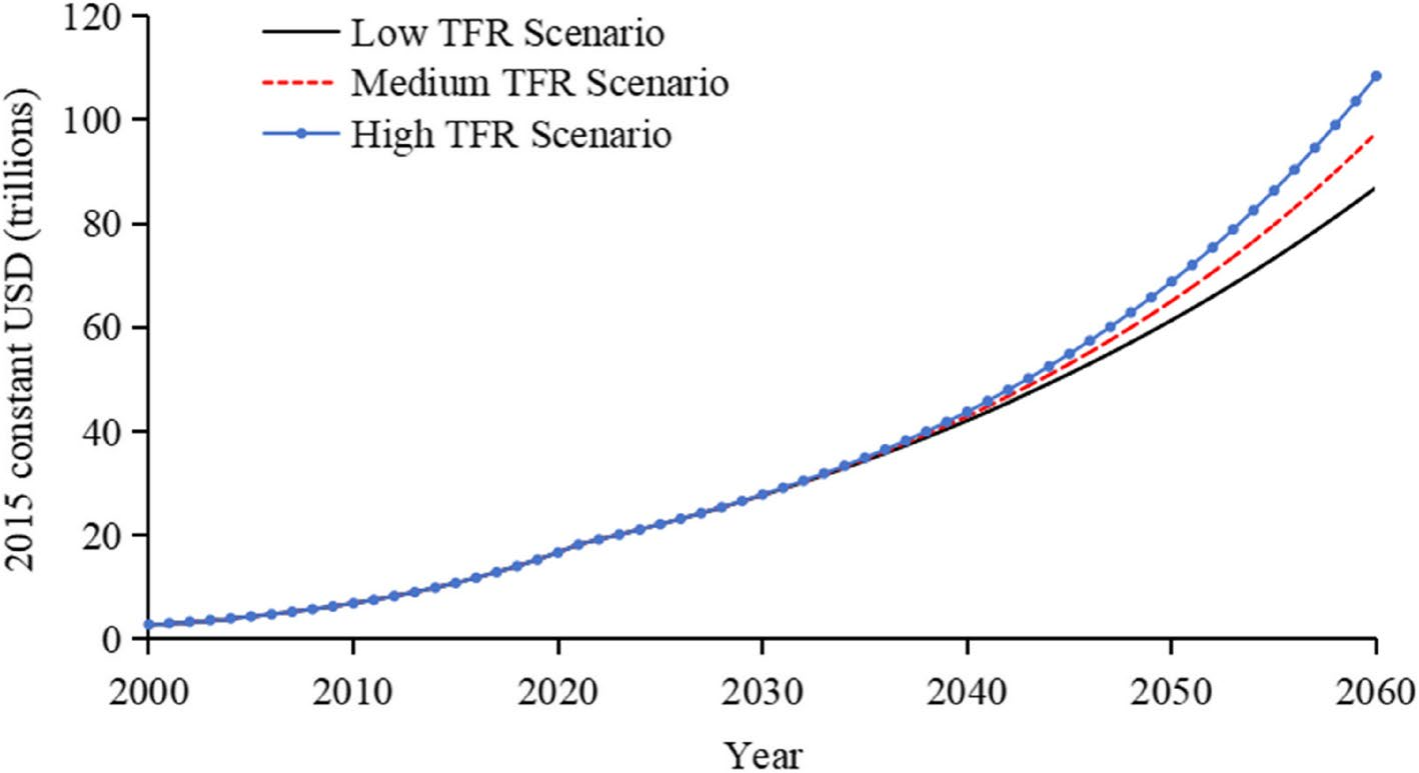
GDP forecast results under different TFR scenarios. Note: Calibration results for China’s total GDP model are shown in Figure A8

All three scenarios exhibit a sustained upward trend in GDP forecasts as shown in Fig. 7. These scenarios begin to diverge starting in 2035 and progressively widen thereafter. This divergence is attributable to the following: during the 2020–2035 period, although the number of newborns varies among the different TFR scenarios, their impact on the workforce and GDP is not yet pronounced, resulting in no significant effects on GDP; during the 2035–2060 period, as these newborns reach working age and enter the workforce, they begin to have a significant positive impact on GDP. Higher TFR scenarios lead to an expanded working-age population and workforce, indicating that demographic shifts, particularly in the proportion of the working-age population, carry significant economic implications.

### The total health expenditure as a percentage of GDP

**Fig. 8:**
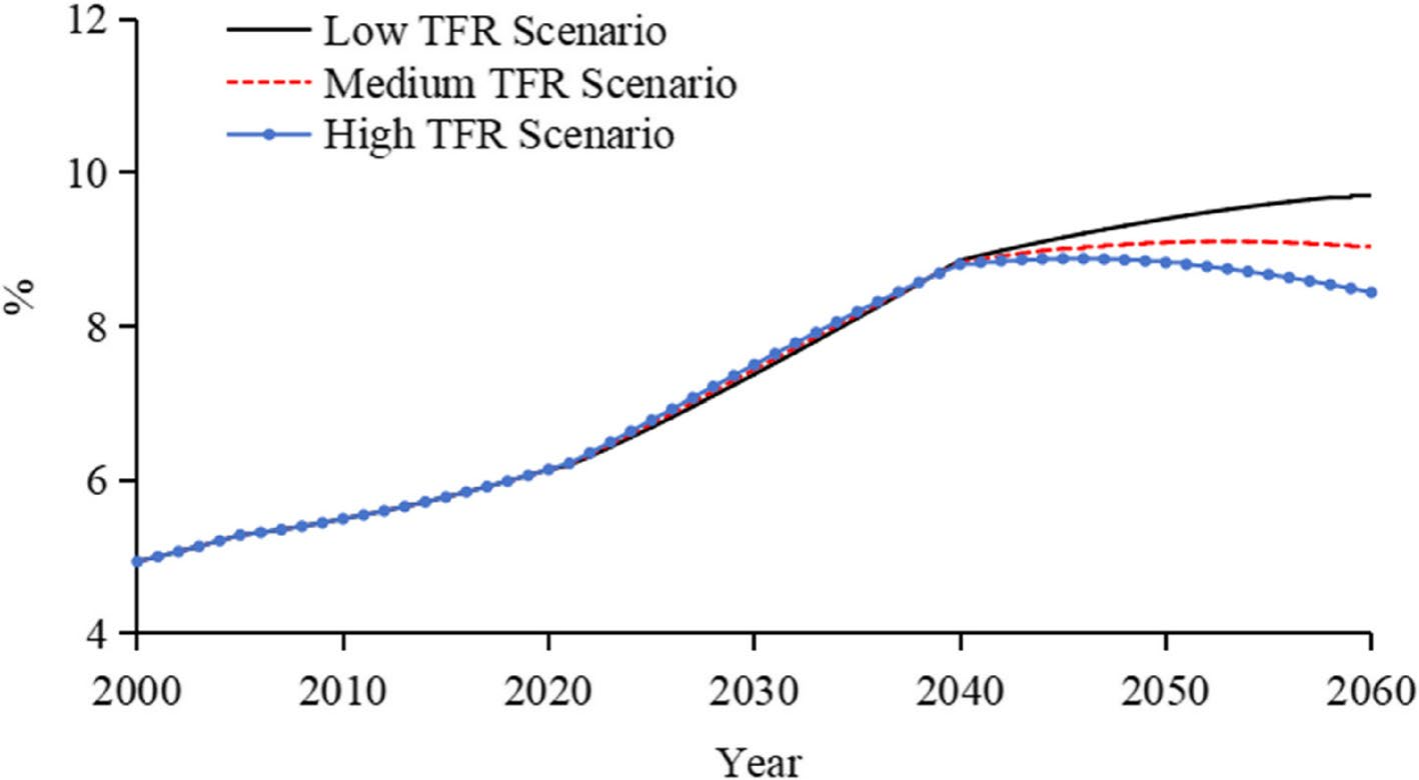
Forecasted Trends of China’s THE as a Percentage of GDP under different TFR scenarios. Note: To calculate the THE/GDP ratio, we employ a low workforce participation rate (refer to Table A6). The efficiency impact factor is set at 3% from 2021 to 2040 and increases to 4% for the period from 2041 to 2060 (see subsequent sections). THE Model (Fig. 1) calculates the THE, and Total GDP Forecasting Model (Fig. 2) computes the GDP, which together allow for the derived Forecasted Trends of China’s THE as a Percentage of GDP, as shown in Fig. 8

During the 2020–2040 period, the THE/GDP ratio remains relatively consistent across all TFR scenarios as shown in Fig. 8. During this period, although the number of newborns varies among the different TFR scenarios, their impact on the workforce and GDP is not yet pronounced, resulting in no significant effects on GDP. Simultaneously, THE may rise due to these newborns, thereby leading to an increase in the THE/GDP ratio. During the 2040–2060 period, as these newborns reach working age and enter the workforce, they begin to have a significant positive impact on GDP, and the THE/GDP ratios start to diverge among the three scenarios. Under the high TFR scenario, an increased workforce drives economic growth, causing GDP growth to outpace the increase in Total Health Expenditure, which leads to a decrease in the THE/GDP ratio. Conversely, under the low TFR scenario, rapid population decline and intensified ageing result in THE growing faster than GDP, thereby causing the THE/GDP ratio to rise. By 2060, under the low, medium, and high TFR scenarios, the THE/GDP ratios are projected to reach around 9.7%, 9.0%, and 8.4%, respectively.

### Total health expenditure adjusted by the efficiency impact factor

The forecasted results under the low TFR scenario of 1.05 indicate that by 2060, THE is expected to be approximately 33.4 trillion USD, with the per capita Health Expenditure expected to reach roughly 30,800 USD as illustrated in Fig. 3. This substantial figure is primarily driven by the increasing growth rates of GDP, THE, and per capita Health Expenditure. To more reasonably quantify and forecast Health Expenditure, we incorporated the concept of potential efficiency improvements as discussed in some research [33–36]. Accordingly, we have adopted an anticipated efficiency gain of 3% per year from 2021 to 2040, 4% per year from 2041 to 2060, adjusting the THE model. Overall, the efficiency factor is arbitrary and calls for empirical investigation in future research. After adjustment, the projected THE for 2060 decreases to around 8.6 trillion USD, and the per capita Health Expenditure decreases to nearly 7,900 USD as illustrated in Table 1.

**Table 1:** Comparative analysis of health expenditure under three TFR scenarios with adjusted efficiency impact factors

Year	Population Size	Population Structure	Projected Health Expenditure	
	**Total Population, in billions**	**Proportion of the Population aged 65 + , %**	**Per capita Health Expenditure in constant 2015 USD**	**Total Health Expenditure in constant 2015 trillion USD**
2000	1.243	7.1	102	0.13
2060 Low TFR Scenario	1.09	34.5	7900	8.6
2060 Medium TFR Scenario	1.22	30.8	7300	8.9
2060 High TFR Scenario	1.36	27.4	6700	9.0

The efficiency impact factor is 3% annually from 2021 to 2040, and 4% annually from 2041 to 2060. SD models emphasize behavioral trends and structural dynamics over exact predictions. Based on this, the precision of forecasted values has been adjusted and appropriately rounded

**Fig. 9:**
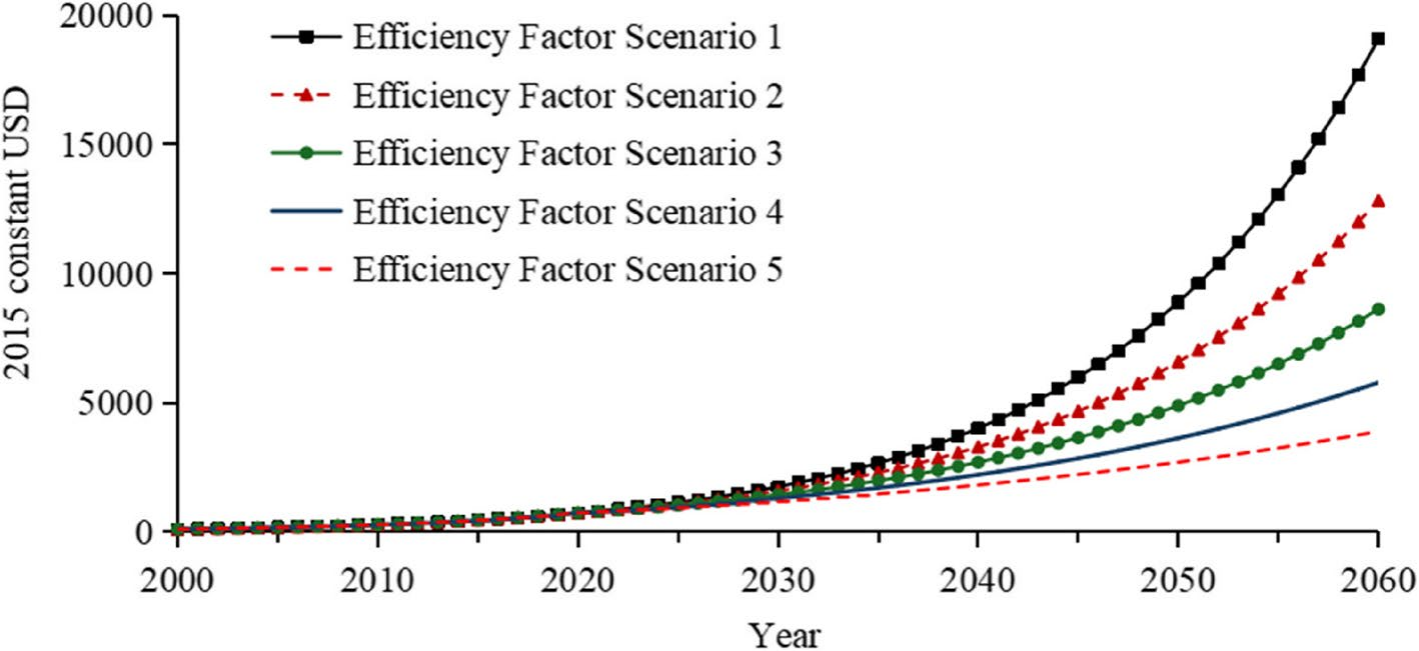
Health Expenditure per capita influenced by various efficiency factors. Note: The Stock-Flow Diagram of the Efficiency Impact Factor is displayed in Figure A5

Figure 9 presents a sensitivity analysis of five efficiency impact factors under a medium TFR of 1.45: annual adjustments in efficiency impact factors from 1 to 5% result in changes to the per capita Health Expenditure in 2060, ranging from approximately 3,900 USD to 19,100 USD in 2015 constant USD. This analysis reveals the potential impact of efficiency adjustments on future health expenditure.

### Health expenditure under three fertility rate scenarios

In our scenario analysis, as the TFR increases, the growing population size and decreased ageing rate are expected to lead to a rise in THE and a decrease in per capita Health Expenditure.

## Discussion

### Main findings

We developed THE SD models and a GDP model to forecast China’s THE and GDP from 2000 to 2060. The forecast shows that with rising TFR, an increase in population size, and a lower ageing rate, THE would rise, while per capita Health Expenditure would decrease.

China’s THE is expected to grow significantly in the future. Under the low TFR scenario, THE is projected to reach around $2.7 trillion by 2030 in 2015 constant USD, increase to about $6.6 trillion by 2040, rise further to about $15.3 trillion by 2050, and reach approximately $33.4 trillion by 2060. After taking into account efficiency factors, THE in 2060 is expected to drop to nearly $8.6 trillion. Total population is projected to gradually decrease from 1.243 billion in 2000 to nearly 1.09 billion by 2060.

Per capita Health Expenditure under the low TFR scenario is expected to rise steadily, from $102 in 2000 to around $30,800 in 2060. When efficiency factors are introduced, per capita Health Expenditure in 2060 is expected to decrease to almost $7,900.

Under the medium TFR scenario, adjustments in the efficiency factor (ranging from 1 to 5% per year) would significantly impact per capita Health Expenditure in 2060, with projected values ranging from almost $3,900 to $19,100.

Under the high TFR scenario, population decline is slower, and the ageing process is relatively mild, leading to more stable growth in the Health Expenditure Index. In contrast, under the low TFR scenario, although ageing is more pronounced, the sharp decrease in population size leads to Total Health Expenditure Index peaking earlier.

Under the high TFR scenario, the population structure would undergo significant changes, with an increase in the working-age population and workforce, which would have a profound impact on GDP growth. This shift would cause GDP to grow faster than THE, resulting in a decrease in the THE/GDP ratio. In comparison, under the low TFR scenario, the rapid decline in population size and accelerated ageing would lead to THE growing faster than GDP, resulting in an increase in the THE/GDP ratio.

### Comparative analysis of China’s GDP forecasting results

As the COVID-19 pandemic subsides, China's economy is gradually recovering, showing a positive resurgence. Despite fluctuations, future growth is expected to be robust. It has been noted that between 2024 and 2035, China’s economic growth potential could reach 8%, with expected growth rates of 5% to 6% [37, 38]. Additionally, it is forecasted that from 2021 to 2030, China’s annual GDP growth rate will be about 5.3%; by 2035, the annual average growth rate will be 5.0%; from 2031 to 2040, it will be 4.4%; and from 2041 to 2050, it will decrease to 3.8% [29].

The World Bank predicts that China’s GDP growth rates in 2024 and 2025 will be 4.6% and 4.4%, respectively [39]. The PricewaterhouseCoopers (PwC) report states that by 2050 China will become the world’s largest economy with a GDP of $58.5 trillion calculated in 2016 constant international dollars, and an annual growth rate of 3.0% [40]. The Organisation for Economic Co-operation and Development (OECD) forecasts that by 2060, the global GDP will be $418 trillion (2014 PPP), with China accounting for 18%, or about $75.24 trillion [41].

Our study, employing a System Dynamics model, analyzes China’s GDP growth from 2021 to 2060 under low, middle, and high TFR scenarios. From 2021 to 2040, the annual growth rates under different TFR scenarios are approximately 4.3%, 4.4%, and 4.5%, respectively; from 2041 to 2060, they are approximately 3.5%, 4.0%, and 4.4%. By 2060, the forecasted GDP is approximately $87 trillion, $97.5 trillion, and $108.4 trillion, calculated in 2015 constant USD, respectively.

This research supplements other economic forecasts by analyzing China’s long-term GDP growth using a System Dynamics model, offering valuable insights for policymakers.

### Comparative analysis of China’s THE forecasting results

China’s THE in 2021 was 7.68 trillion yuan in current prices, accounting for 6.72% of GDP [23]. It is predicted that by 2025, THE could reach 8.92 trillion yuan in current prices, representing 6.37% of GDP [13]. Another forecast estimates that THE might reach 21.39 trillion yuan by 2030, with a GDP share of 14.49% [42]. It is also predicted that China’s THE could be 9.45 trillion yuan in current prices in 2022 [14]. In comparison, our study, using a current-price model, forecasts that nominal THE is expected to reach approximately 11.6 trillion yuan by 2025, accounting for about 6.2% to 7.8% of GDP. By 2030, THE is forecast to potentially increase to nearly 21.4 trillion yuan, accounting for 6.5% to 10.7% of GDP. Detailed differences among the forecasts can be found in Table A4.

### Relationship between per capita health expenditure and health-adjusted life expectancy

Between 2001 and 2019, China’s Current Health Expenditure per capita, PPP (current international $) increased significantly from 135.9 to 885.9, while Health-Adjusted Life Expectancy (HALE) improved from 63.7 to 68.5 years, reflecting the positive impact of increased Health Expenditure on HALE, as shown in Figure A10. This trend presents important role of Health Expenditure in improving HALE outcomes. However, when looking at different time horizons, the strength of the relationship appears to vary. For instance, the short-term data (2000–2022) shows a strong correlation (R^2^ = 0.8388), while the long-term data (2000–2060) reveals a weaker correlation (R^2^ = 0.5307). This discrepancy in correlations could be due to the diminishing returns on life expectancy as Health Expenditure increases—initial gains are significant, but the effect gradually diminishes as HALE reaches higher levels. Additionally, the burden of chronic diseases associated with an ageing population further weakens the impact of Health Expenditure on life extension [43–45].

### Scenarios consideration

Our System Dynamics (SD) models include three TFR scenarios: low (1.05), medium (1.45), and high (1.85). These scenarios are somewhat conservative and may underestimate the impact of uncertainties in the model. To address this, we have provided an interactive version of the simulation model online at this link: https://exchange.iseesystems.com/public/simulation-results/bmc-paper-simulation-results. This platform allows readers to experiment with their own scenarios using adjustable variable sliders. TFR can be adjusted between 1.0 and 3.0. Retirement trends are modeled with three scenarios for workforce participation rates: normal (0), delayed retirement (1), and maximum delayed retirement (2). Efficiency adjustment factors range from 0 to 5% per year, while GDP productivity growth can be adjusted from 1 to 9% annually. By allowing such adjustable parameters, the model aims to address uncertainties and provide flexible projections under varying assumptions.

### Limitations

Our THE SD models focus on ageing, annual health demand growth, and facility and pharmaceutical cost growth, but do not account for other factors influencing Total Health Expenditure. We do not incorporate endogenous feedback mechanisms (with the exception of population growth) from System Dynamics. Instead, we rely more on calculations and forecast related to stocks and flows in System Dynamics. Furthermore, we utilize Australia’s per capita grouped Health Expenditure to forecast China’s Total Health Expenditure, which might not be perfectly aligned because of differences in ageing trends and healthcare systems, among other factors. Also, incorporating per capita grouped Health Expenditure from other high- and middle-income countries for sensitivity analysis would be beneficial, which presents a valuable opportunity for future research. Despite our efforts to design comprehensive scenarios, they remain relatively conservative and may not fully capture the inherent uncertainties in such projections. Future research could expand the scope and consider alternative scenario designs to better reflect the diversity and complexity of these dynamics. Finally, societal ageing is a global issue that affects many countries, not just China. However, this paper primarily focuses on the impact of China’s ageing population on THE.

### Enhancing the efficiency of health expenditure

Facing growing fiscal pressures, it is vital to adopt diversified strategies to enhance the efficiency of health care funding. This requires a deep consideration of the specific needs of different regions and populations, strengthening the linkage between input and output, and continually optimizing the mechanisms for budgeting, fund utilization, and performance assessment of both regular and special health funds. Optimization across multiple dimensions, including preventive health care and the operational management of medical institutions, can effectively improve the efficiency of government health funding and enhance the well-being of residents [13].

### Enhancing TFR policies

Increasing the TFR can help alleviate population decline and ageing to some extent. Developing a comprehensive, systematic, and sustainable TFR support policy system is crucial for increasing TFR in response to changes in the macro-fertility environment. Implementing policies in areas such as employment, taxation, healthcare, and household consumption can reduce housing and rental costs for people of childbearing age and increase public education investment. Building a TFR support policy system and increasing TFR is a long-term process that requires support for all children, including families with one or two children [46]. The goverment can further enhance measures for the three-child policy by including childcare expenses for children under three in tax deductions and expanding universally accessible childcare services, thus easing family pressures related to childbirth, upbringing, and education [47].

### Enhancing workforce productivity

As China’s population ages, workforce shortages are becoming increasingly severe. China’s domestic policy reforms aimed at boosting productivity potential are expected to support economic growth for decades to come [7, 48]. Automation offers a potential solution to improve workforce productivity, while also driving advances in education and economic growth. Enhancing the quality of children’s education can strengthen their competitiveness and raise overall workforce productivity. Moreover, artificial intelligence has the potential to reduce skill premiums and enhance overall workforce productivity, making it better equipped to address the challenges of an ageing population and promote economic development [49–51].

### Maintaining stable economic growth is essential

Sustained economic growth can be very helpful to counteract the challenges posed by an ageing population. This growth plays a crucial role in offsetting the escalating costs associated with demographic ageing [43, 52]. The provision of financial resources and social services is essential to support older adults who face Catastrophic Health Expenditure either regularly or intermittently, thereby enhancing their health outcomes [53]. It is also critical to foster policy synergies across economic, social, and health sectors. Such concerted efforts are fundamental in enhancing the quality of life for the ageing population and ensuring the economy’s sustainable development [54]. Additionally, improving access to health opportunities is crucial. Health opportunities refer to the potential and likelihood for individuals from different socioeconomic classes to access health resources and services [55]. These involve the conditions that promote, maintain, or improve health at both individual and collective levels. This approach not only strengthens human capital but also boosts productivity, contributing significantly to the overall economic performance [56–58].

## Conclusion

This study is the first to utilize Australia’s per capita Health Expenditure by age and sex to create a per capita Health Expenditure index by age and sex tailored to China. We combine this index by age and sex with the Array Population Model of China to build two THE SD models to forecast China’s THE and Health Expenditure by age and sex from 2000 to 2060. In addition, we developed a new Total GDP Forecast Model to estimate THE/GDP. Overall, our models provide a useful tool for analyzing future Total Health Expenditure and demographic trends in China. The total GDP forecast model, while secondary, helps estimate THE/GDP, providing a clearer view of the economic impact of population ageing and Total Health Expenditure, thereby supporting health policy decisions.

## Supplementary Information

Additional file 1. Appendices (figures and tables). For detailed system dynamics simulation results, please refer to the following link: https://exchange.iseesystems.com/public/simulation-results/bmc-paper-simulation-results.

## Data Availability

The datasets analysed during the current study are available from the corrsponding author on reasonable request, and permission was given for the access.

## Abbreviations

THE: Total Health Expenditure
HE pc: Health Expenditure per capita
SD: System Dynamics
SFD: Stock-Flow Diagram
GDP: Gross Domestic Production
USD: United States Dollars
TFR: Total Fertility Rate
HALE: Health-Adjusted Life Expectancy
DALYs: Disability-adjusted Life Years
GBD: Global Burden Disease
PPP: Purchasing Power Parity
BRICS: Brazil, Russia, India, China, South Africa
OECD: Organisation for Economic Co-operation and Development
PwC: PricewaterhouseCoopers
