# Supplementary material for: Forecast of total health expenditure on China’s ageing population: a system dynamics model
Source: BMC Health Serv Res. 2024 Dec 27;24:1655. doi: 10.1186/s12913-024-12113-6 (PMC11681677; doi:10.1186/s12913-024-12113-6)
Supplement: Supplementary file 1 — Additional file 1. Appendices (figures and tables). For detailed system dynamics simulation results, please refer to the following link: https://exchange.iseesystems.com/public/simulation-results/bmc-paper-simulation-results. [file 12913_2024_12113_MOESM1_ESM.docx]

**Appendix**

**Table A1** Key Variables of the Total Health Expenditure Model

| **Variable Name** | **Variable Type** | **Definition** | **Unit** |
| --- | --- | --- | --- |
| Population by Age and Sex (Array Population Model) | Shadow Variable | Derived from the Array Population Model [18], it serves as a "shadow variable" representing the population divided by age and sex (in five-year groups). | Million People |
| Per Capita Health Expenditure Group Index | Array Auxiliary Variable | This study introduces a new metric, the Per Capita Health Expenditure Group Index, based on Australia’s per capita Health Expenditure. This index categorizes China’s Health Expenditure by age and gender, providing a basis for analysis of per capita Health Expenditure. | – |
| Health Expenditure Group Index | Array Auxiliary Variable | The Health Expenditure Group Index is calculated by combining population groups by age and sex (every five years) to determine the average Health Expenditure for each group. This index, based on the concept of group summation, reveals the Health Expenditure of different groups. | – |
| Per Capita Health Expenditure Index | Auxiliary Variable | The Per Capita Health Expenditure Index reflects the per capita Health Expenditure in an index form. | – |
| Health Expenditure Per Unit Index | Auxiliary Variable | Health Expenditure Per Unit Index. | – |
| Health Demand Growth Index | Stock | The Health Demand Growth Index aims to reflect the growing trend of healthcare expectations in the modern population. Consistent with the model assumptions, it is used as an adjustment factor in the forecast model of China's Total Health Expenditure. Its validity will be verified through model validation. | – |
| Actual Cost Growth Index of Medical Services | Stock | The Actual Cost Growth Index of Medical Services is designed to reflect the actual increase of the real cost of medical facilities and pharmaceuticals. Similar to the Health Demand Growth Index, it aligns with the model’s assumptions and serves as an adjustment factor in the forecast model of China’s THE. Its accuracy will be confirmed through subsequent model calibration. | – |
| Total Health Expenditure of China | Auxiliary Variable | Total Health Expenditure describes the total amount of economic resources gathered in a country during a year to support various health service activities. | 2015 Constant USD or Current Price CNY |
| Per Capita Health Expenditure | Auxiliary Variable | Per Capita Health Expenditure refers to the ratio of Total Health Expenditure in a year to the total population in that year. | 2015 Constant USD or Current Price CNY |
| Gross Domestic Product (GDP) | Stock | Gross Domestic Product (GDP) measures the total market value of all final goods and services produced within a country or region over a certain period. | 2015 Constant USD or Current Price CNY |
| Total Health Expenditure as a Percentage of GDP | Auxiliary Variable | The ratio of Total Health Expenditure to GDP represents the proportion of Health Expenditure to the Gross Domestic Product (GDP) in a specific year. This ratio reveals the economic commitment to healthcare and the overall attention to public health and well-being by the government and society during a period. | % |

**Table A2** Variables of the Total GDP Forecasting Model

| **Variable Name** | **Variable Type** | **Definition** | **Unit** |
| --- | --- | --- | --- |
| Forecast Total GDP | Auxiliary Variable | A projected simulation of GDP from 2000 to 2060, based on workforce size, initial GDP per worker, and the per capita GDP productivity index of the workforce. | 2015 constant USD |
| Workforce | Shadow Variable | Sum of all Grouped Workforces, where each Grouped Workforce refers to the product of the Grouped Population and their corresponding workforce participation rates. | Million People |
| Initial GDP Per Workforce | Auxiliary Variable | The per capita GDP of the workforce in the year 2000. | 2015 constant USD |
| Per Capita GDP Productivity Index of the Workforce | Stock | The accumulated per capita productivity of the workforce for the specified year. | – |
| Changes in Workforce Productivity | Flow | Reflects the annual changes in workforce productivity. | – |
| Productivity Index Growth Rate | Auxiliary Variable | Productivity Index Growth Rate refers to the annual growth rate of the Per Capita GDP Productivity Index of the Workforce from 2000 to 2060. It is defined as a 9% growth rate from 2000 to 2019, and a 5% growth rate for the period post-2020. | % |
| Post-2020 Productivity Index Growth Rate | Auxiliary Variable | Post-2020 Productivity Index Growth Rate refers to the annual growth rate of the per capita GDP Productivity Index of the Workforce from 2020 to 2060. The productivity index growth rate is set at 5% for the period post-2020. | % |

**Table A3** Simulation Equations for the Model’s Main Variables

| **Variable Name** | **Simulation Equation** |
| --- | --- |
| Total Health Expenditure of China (t) | Health Expenditure Per Unit Index (t) * Total Health Expenditure Index (t) * Health Demand Growth Index (t) * Actual Cost Growth Index of Medical Services (t) |
| Health Expenditure Per Unit Index (t) | INIT(Total Health Expenditure of China (Historical Data) / Total Health Expenditure Index (t)) |
| Total Health Expenditure Index (t) | SUM(Health Expenditure Group Index (t)) |
| Health Demand Growth Index (t) | Health Demand Growth Index (t - dt) + (Increment of Health Demand Index) * dt |
| Per Capita Health Expenditure Index (t) | Total Health Expenditure Index (t) / Total Population (t) |
| Actual Cost Growth Index of Medical Services (t) | Actual Cost Growth Index of Medical Services (t - dt) + (Increment of Medical Cost Index) * dt |
| Total Population (t) | SUM(Grouped Population (t) (Array Population Model)) |
| Forecast Total GDP (t) | Workforce * Initial GDP per worker *per capita GDP Productivity Index of the Workforce |
| Workforce | SUM(Grouped Workforce) |
| Grouped Workforce | Grouped Population *Grouped Workforce Participation Rate |

**Table A4** Comparison of Research Findings on the Forecast of China’s Total Health Expenditures

| Selected Studies | Zheng et al. (2020) [*] | | Feng Yuanyuan (2022) [*] | Liu Qiaoyan (2018) [*] | Our Study (2024) |
| --- | --- | --- | --- | --- | --- |
| Model | ARIMA | | ARIMA | SD | SD |
| Simulated Indicator | THE | | THE | THE | THE |
| Simulation Period | 1978-2022 | | 2000-2030 | 2001-2025 | 2000-2060 |
| Influencing Factors | — | | The proportion of the population aged 65 and above, per capita GDP, urban-rural income gap, basic medical insurance expenditure for urban and rural residents, the number of healthcare workers per 1,000 people, and the number of hospitals, etc. | Population size, elderly population, GDP, Government Health Expenditure, healthcare professionals per 1,000 people, and drug costs | Population size, population ageing, actual costs of medical services, and health demand |
| Unit | Current prices (RMB) | | Current prices (RMB); Constant prices (2000 RMB) | Current prices (RMB) | Current prices (RMB); Constant 2015 US$ |
| THE | Current prices | 9.45 trillion RMB (2022) | 21.39 trillion RMB (2030 ) | 8.92 trillion RMB (2025 ) | 8.00 trillion RMB (2022);  11.61 trillion RMB (2025);  21.38 trillion RMB (2030) |
|  | Constant prices | — | 10.19 trillion RMB (2030) | — | 33.43 trillion US$ (2060)  8.55 trillion US$ (2060, after adjusting for efficiency factors) |
| THE pc | Current prices | — | 14,578 RMB (2030 ) | 5,797 RMB (2025 ) | 5,741 RMB (2022 )  8,350 RMB (2025 )  15,569 RMB (2030 ) |
|  | Constant prices | — | 7,270 RMB (2030) | — | 30,816 US$ (2060 );  7,884 US$(2060 , after adjusting for efficiency factors) |
| THE/GDP | 8.20% (2022) | | 14.49% (2030, current prices) | 6.37% (2025) | 9.72% (2060, after adjusting for efficiency factors) |

**Note:** THE pc refers to Per Capita Total Health Expenditure. The data from our study in the table are based on the low TFR scenario. RMB refers to Chinese yuan current prices.

We employed Australian actual dollar values to develop a relative index of per capita Health Expenditure by age and sex, as shown in Table A5. This index reflects the proportion of per capita health expenditures across different age groups, correlating directly with the magnitude of these expenditures.

**Table A5** Health Expenditure Per Capita by Age and Sex Index

| **Age Group** | **Male** | **Female** |
| --- | --- | --- |
| <5 years | 1.4 | 1.142 |
| 5-9 years | 0.3 | 0.3 |
| 10-14 years | 0.3 | 0.3 |
| 15-19 years | 0.5 | 0.65 |
| 20-24 years | 0.61 | 0.9 |
| 25-29 years | 0.65 | 1.4 |
| 30-34 years | 0.71 | 1.7 |
| 35-39 years | 0.7 | 1.3 |
| 40-44 years | 0.85 | 1 |
| 45-49 years | 1 | 1 |
| 50-54 years | 1.3 | 1.2 |
| 55-59 years | 1.8 | 1.5 |
| 60-64 years | 2.4 | 1.9 |
| 65-69 years | 3.4 | 2.7 |
| 70-74 years | 4.73 | 3.8 |
| 75-79 years | 6.35 | 5 |
| 80-84years | 7.65 | 6.4 |
| 85-89years | 8.9 | 7.5 |
| 90-94years | 9.8 | 8.3 |
| 95-99years | 9.3 | 7.1 |
| 100-104years | 9.3 | 7.1 |

**Data sources:** Australian Institute of Health and Welfare, 2017 and our own calculations

**Note:** We transformed the Health Expenditure Per Capita by Age and Sex from the Australian Institute of Health and Welfare (2017) into a Health Expenditure Per Capita by Age and Sex Index based on the provided proportions.

**Table A6** Age and Sex-specific Workforce Participation Rates Set from 2010 to 2050

| **year** | **Age Group (years)** | | | | | | | | | | |
| --- | --- | --- | --- | --- | --- | --- | --- | --- | --- | --- | --- |
|  | 15-19 | 20-24 | 25-29 | 30-34 | 35-39 | 40-44 | 45-49 | 50-54 | 55-59 | 60-64 | 65+ |
| Male |  |  |  |  |  |  |  |  |  |  |  |
| 2005 | 33.0 | 84.3 | 96.4 | 96.9 | 96.7 | 95.8 | 93.8 | 88.6 | 75.6 | 58.9 | 27.8 |
| 2010 | 25.5 | 81.7 | 96.4 | 96.9 | 96.7 | 95.8 | 93.8 | 88.6 | 75.6 | 58.9 | 23.6 |
| 2015 | 20.0 | 77.7 | 96.4 | 96.9 | 96.7 | 95.8 | 93.8 | 88.6 | 75.6 | 58.9 | 22.6 |
| 2020-2050^a^ | 20.0 | 75.0 | 96.4 | 96.9 | 96.7 | 95.8 | 93.8 | 88.6 | 75.6 | 58.9 | 20.0 |
| 2020-2050^b^ | 20.0 | 75.0 | 96.4 | 96.9 | 96.7 | 95.8 | 93.8 | 90.0 | 85.0 | 65.0 | 20.0 |
| Female |  |  |  |  |  |  |  |  |  |  |  |
| 2005 | 35.4 | 76.8 | 82.0 | 84.1 | 85.7 | 84.6 | 76.4 | 63.9 | 52.1 | 38.8 | 13.8 |
| 2010 | 30.4 | 72.2 | 82.0 | 84.1 | 85.7 | 84.6 | 76.4 | 63.9 | 52.1 | 38.8 | 13.8 |
| 2015 | 25.7 | 70.0 | 82.0 | 84.1 | 85.7 | 84.6 | 76.4 | 63.9 | 52.1 | 38.8 | 13.8 |
| 2020-2050^a^ | 20.0 | 70.0 | 82.0 | 84.1 | 85.7 | 84.6 | 76.4 | 63.9 | 52.1 | 38.8 | 10.0 |
| 2020-2050^b^ | 20.0 | 70.0 | 82.0 | 84.1 | 85.7 | 84.6 | 76.4 | 75.0 | 60.0 | 50.0 | 10.0 |

**Note:** Due to space limitations, changes in workforce participation rates between years are not shown here.

**Reference:** Ma Z, Lv Z, Ye K. Labor participation rate and labor force growth: 1982-2050. *Chin J Popul Sci*. 2010;1:11–27, 111. (In Chinese).

**Table A7** List of Abbreviations

| **Abbreviation** | **Full Term** | **Definition** | **Source** |
| --- | --- | --- | --- |
| THE | Total Health Expenditure | Total Health Expenditure includes both public and private spending on health services, such as preventive and curative care, family planning, nutrition programs, and health-related emergency aid. It excludes expenditures on water and sanitation. | World Bank |
| HE pc | Health Expenditure per Capita | Total Health Expenditure divided by the population. | Our World in Data |
| SD | System Dynamics | System Dynamics is a computer-aided method that uses feedback-based simulation for strategy and policy design | System Dynamics Society |
| SFD | Stock-Flow Diagram | A stock-flow diagram visually represents resource dynamics and interactions in a System Dynamics model. | VENTANA Systems.inc |
| GDP | Gross Domestic Product | GDP sums the gross value added by all resident producers, including product taxes and excluding subsidies, without accounting for asset depreciation or resource depletion. | World Bank |
| USD | United States Dollars | The USD (U.S. Dollar) is the official currency of the United States and a globally dominant medium of exchange, reserve currency, and store of value. | Federal Reserve Board |
| TFR | Total Fertility Rate | The estimated average number of children a woman would have if she lived through her childbearing years following current age-specific fertility rates. | World Bank |
| HALE | Health-Adjusted Life Expectancy | The average years a person can expect to live in full health, accounting for years lost to disease or injury. | World Health Organization |
| DALYs | Disability-adjusted life years | One DALY equals one year of lost full health, combining years lost to premature death (Years of Life Lost; YLLs) and years lived with disability (Years Lived with Disability; YLDs) from a disease or condition. | World Health Organization |
| GBD | Global Burden Disease | The GBD study is the largest effort to measure health loss globally over time, aiming to improve health systems and reduce disparities. | Global Burden Disease |
| PPP | Purchasing Power Parity | PPP compares the purchasing power of currencies by measuring how much of one country’s currency is needed to buy the same goods and services as in another, eliminating price level differences. | World Bank |
| BRICS | Brazil, Russia, India, China, South Africa | BRICS is a group of five emerging economies, Brazil, Russia, India, China, and South Africa, focused on global cooperation and influence. | Instituto de Pesquisa Econômica Aplicada |
| OECD | Organisation for Economic Co-operation and Development | The OECD is an international organisation shaping policies to promote prosperity, equality, and well-being, with over 60 years of expertise. | OECD |
| PwC | PricewaterhouseCoopers | A global professional services network providing audit and consultancy. | PwC |


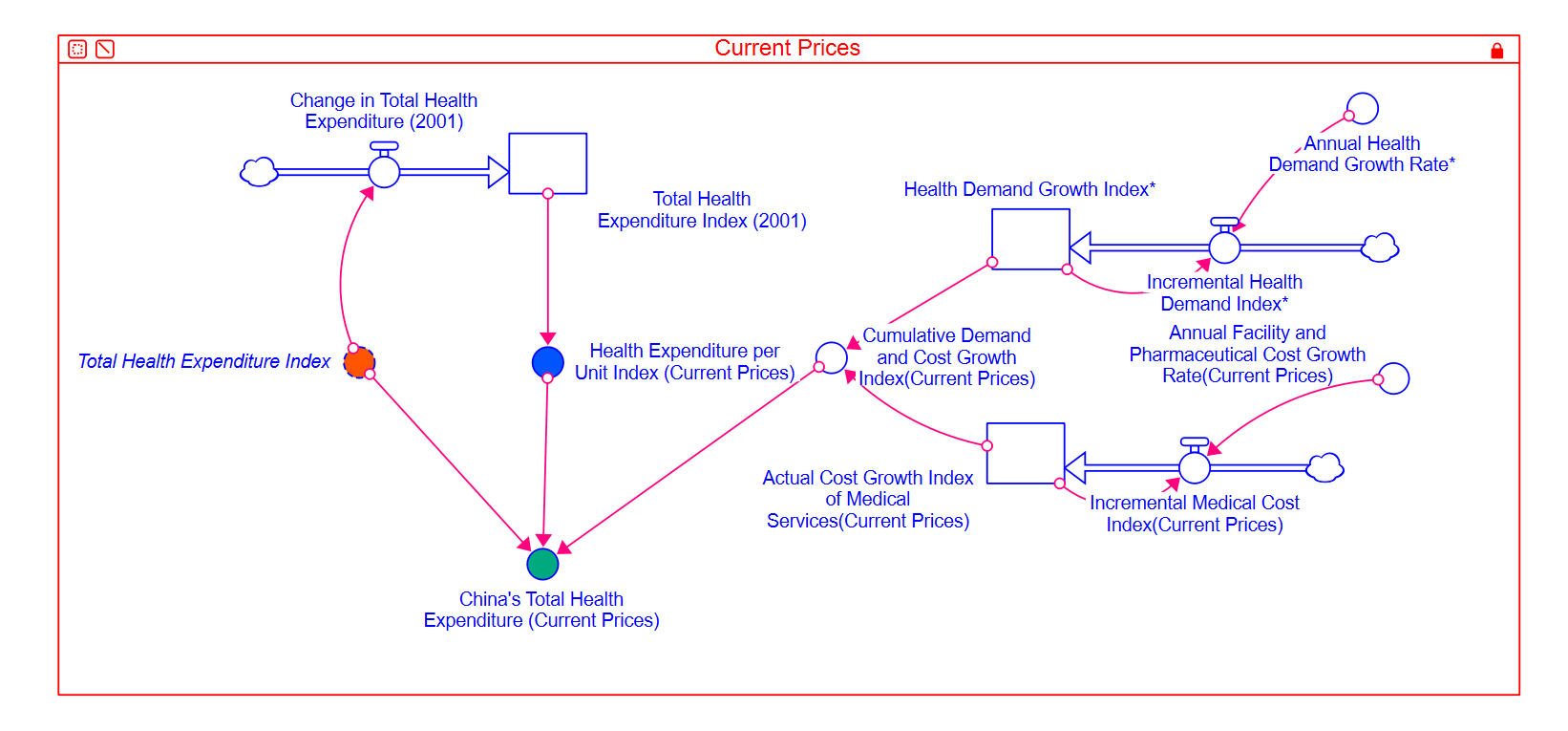


**Figure A1** Stock-Flow Diagram of the Total Health Expenditure Model (Current Prices)

### The calibration results of China’s Total Health Expenditure Model at constant prices, as shown in Fig. A2, demonstrate the THE model’s forecasting capacity when compared with historical data from 2000 to 2021. The THE model exhibits an alignment of 92.5% with the historical data, indicating its considerable forecasting accuracy. This is further corroborated by linear regression analysis, yielding a result of y = 0.8962x with a high correlation coefficient (R²) of 0.9912 (See Figure A2). Such significant statistical correlation suggests the model’s reliability in forecasts and its potential for highlighting trends in THE.

**
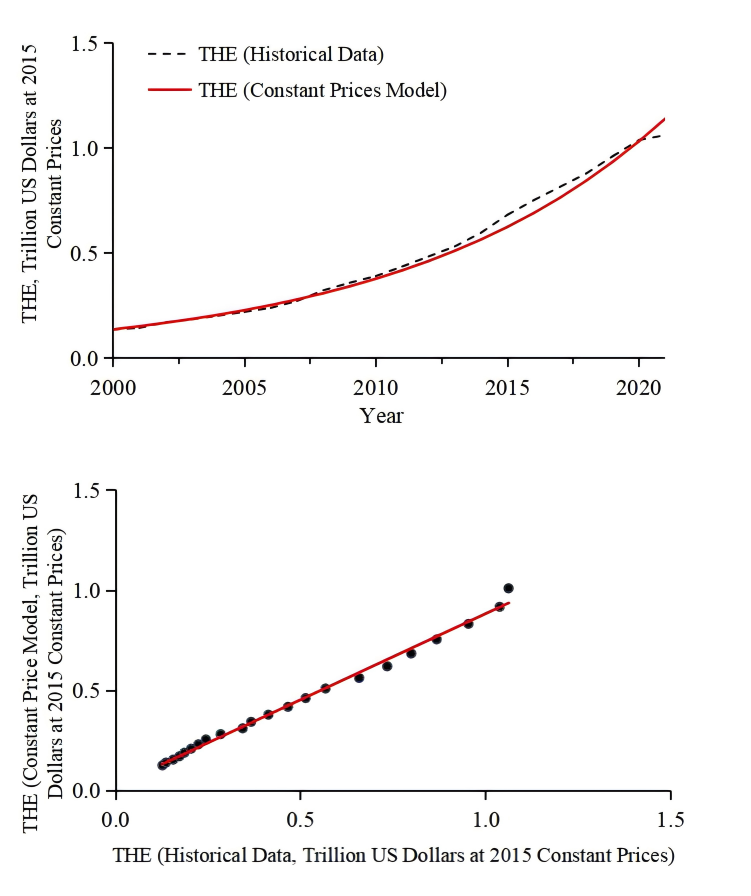
**

**Figure A2** Calibration for China’s Total Health Expenditure Model at Constant Prices

Note: Regression lines are estimated using Ordinary Least Squares (OLS) methodology. The red line represents the linear regression result with a slope of 0.9819 (p-value<0.001). The coefficient of determination, R^2^=0.9912, quantifies the proportion of the variance in China’s Total Health Expenditure (Historical Data) that is explained by China’s Total Health Expenditure (Constant Price Model).


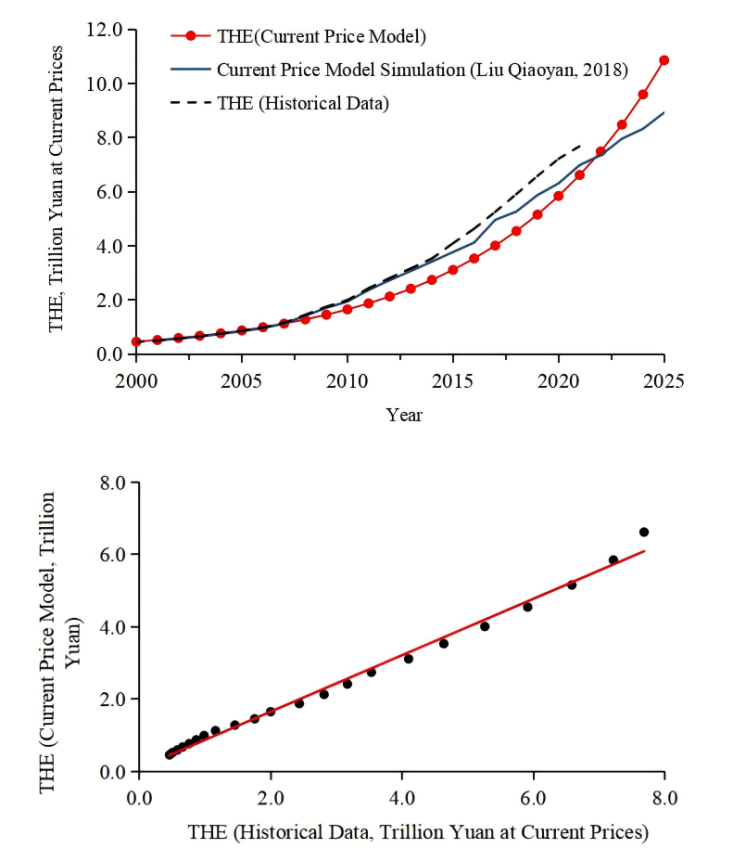


**Figure A3**  Calibration Results for China’s Total Health Expenditure Model at Current Prices

### Note: The unit is in current billion yuan.

### This study primarily employs a constant price model, concurrently verifying it with a current price model to further reinforce its robustness and reliability. We discover that the data provided by the China Statistical Yearbook [22] and the results of Liu [13] both utilize current prices as a benchmark. This model, while considering the constant price basis, incorporates inflation factors (adjusted and calculated based on the World Bank’s Consumer Price Index) [20] and embeds the inflation parameter within the variable of annual healthcare cost growth rates (current prices). Analyzing historical data from 2000 to 2021, the model demonstrated a high reliability and accuracy with an average alignment of 86.2% to actual data. Further calibration through linear regression analysis yielded a result of y = 0.7999x + 1E+11, with a coefficient of determination (R²) of 0.9922, which further attests to the model’s accuracy. Additionally, compared with the simulated Total Health Expenditure of China (2001-2025) by Liu [13], the model achieved an average alignment of 87.3%, indicating its strong forecasting capability. The corresponding linear regression analysis resulted in y = 1.003x, with a coefficient of determination (R²) of 0.9532, corroborating the model’s forecasting power from another perspective. In addition, our results are consistent with Zhai and his colleagues [16].


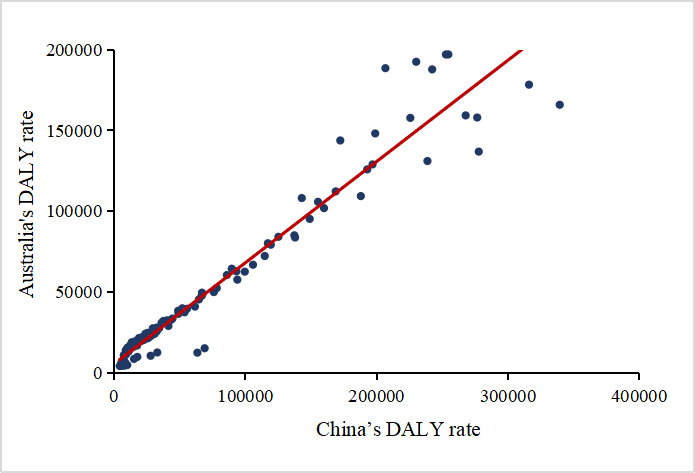


**Figure A4** Comparative Analysis of Age- and Sex-Specific Disability-Adjusted Life Years (DALYs) Rates between China and Australia for the years 2000, 2010, and 2019

**Note:**The unit represents one per 100,000. Regression lines are estimated using Ordinary Least Squares (OLS) methodology. The red line represents the linear regression result with a slope of 0.627 (p-value<0.001). The coefficient of determination, R^2^=0.9424, quantifies the proportion of the variance in China’s DALY rate that is explained by Australia’s DALY rate.


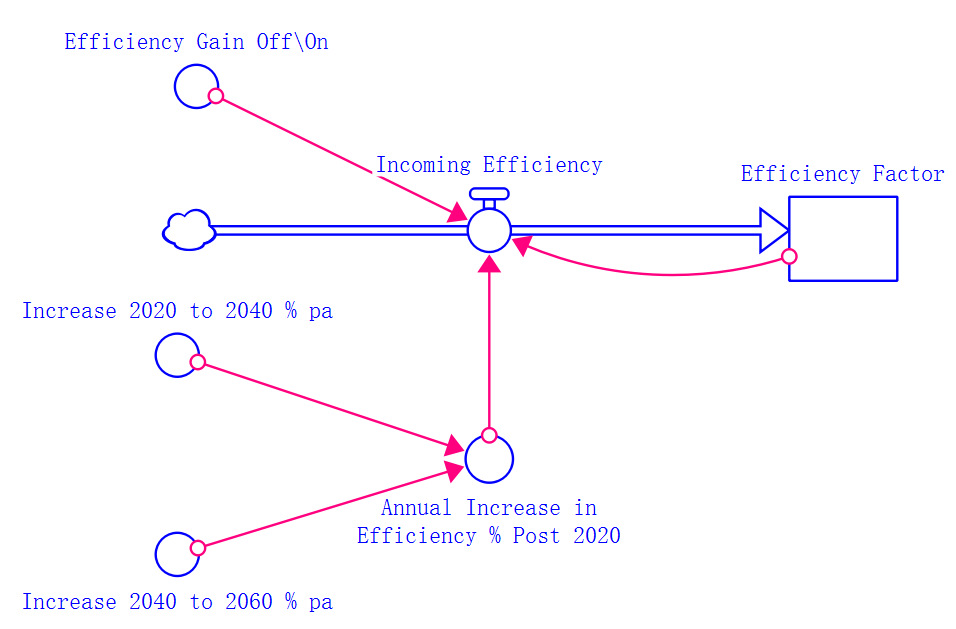


**Figure A5** Stock-Flow Diagram(SFD) of the Efficiency Impact Factor

###
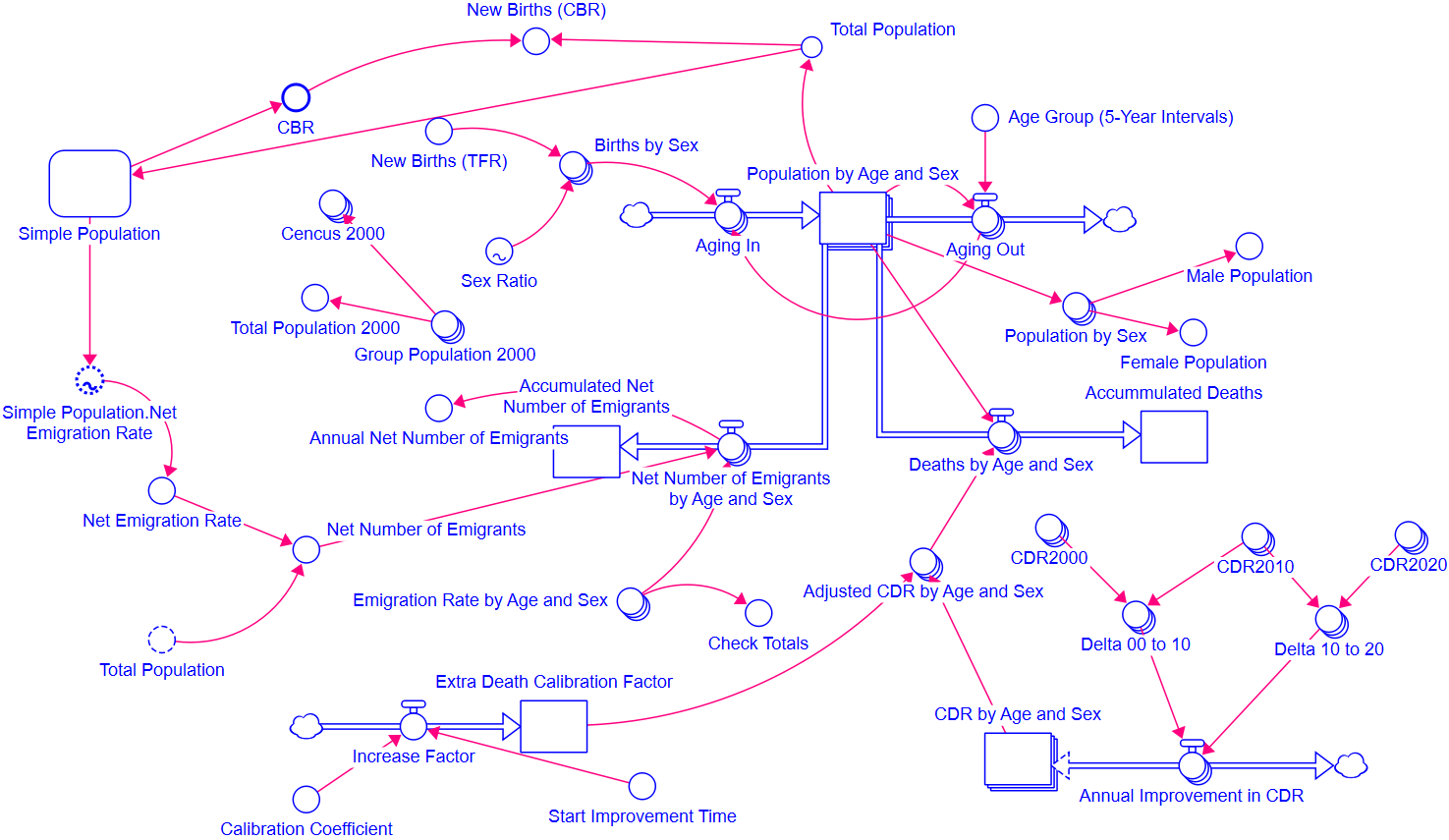


### **Figure. A6** Array Population Model[18]

**Note:** ageing in and ageing out refers to when an individual leaves one age group and enters the next one.


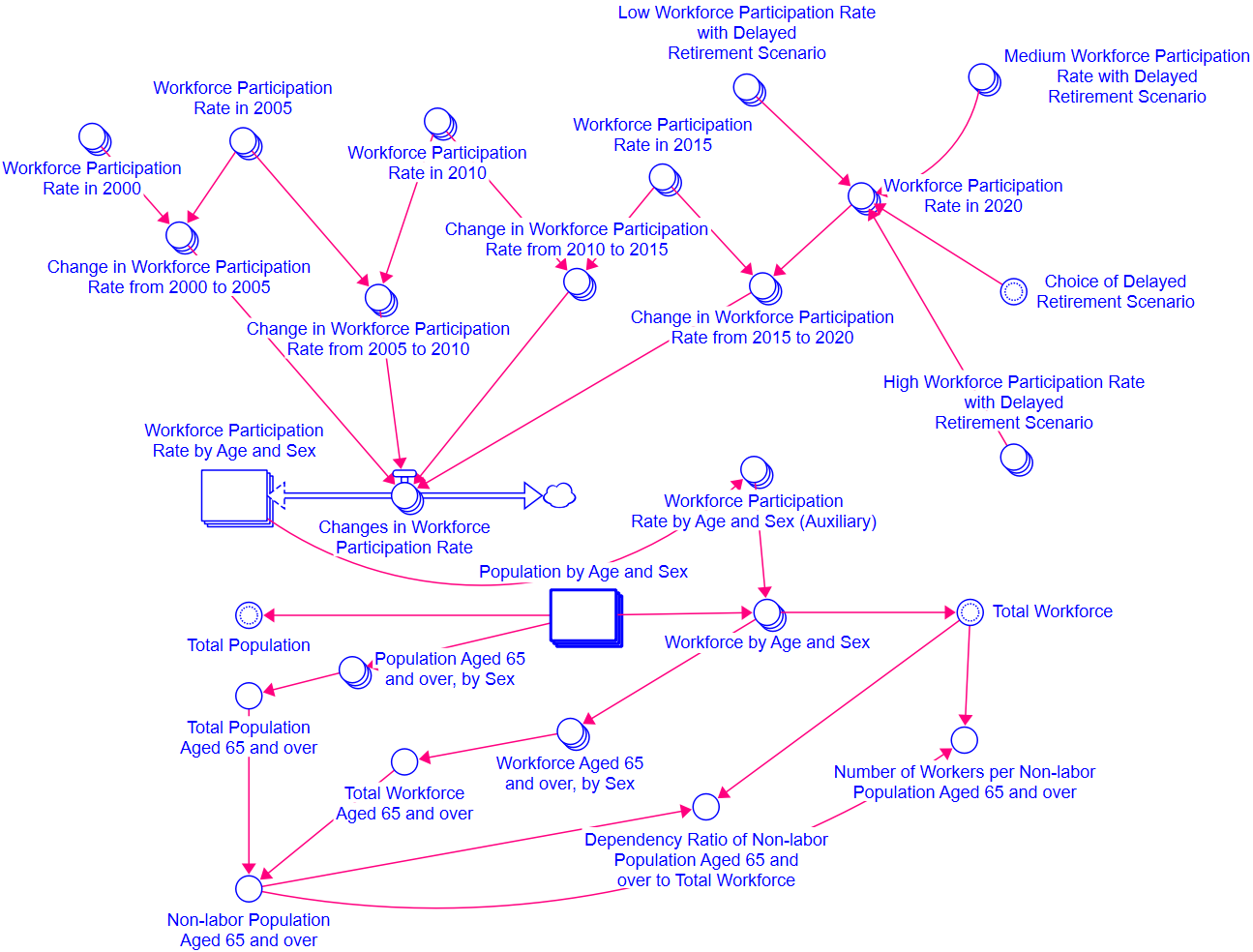


### **Figure. A7** Workforce Model[18]


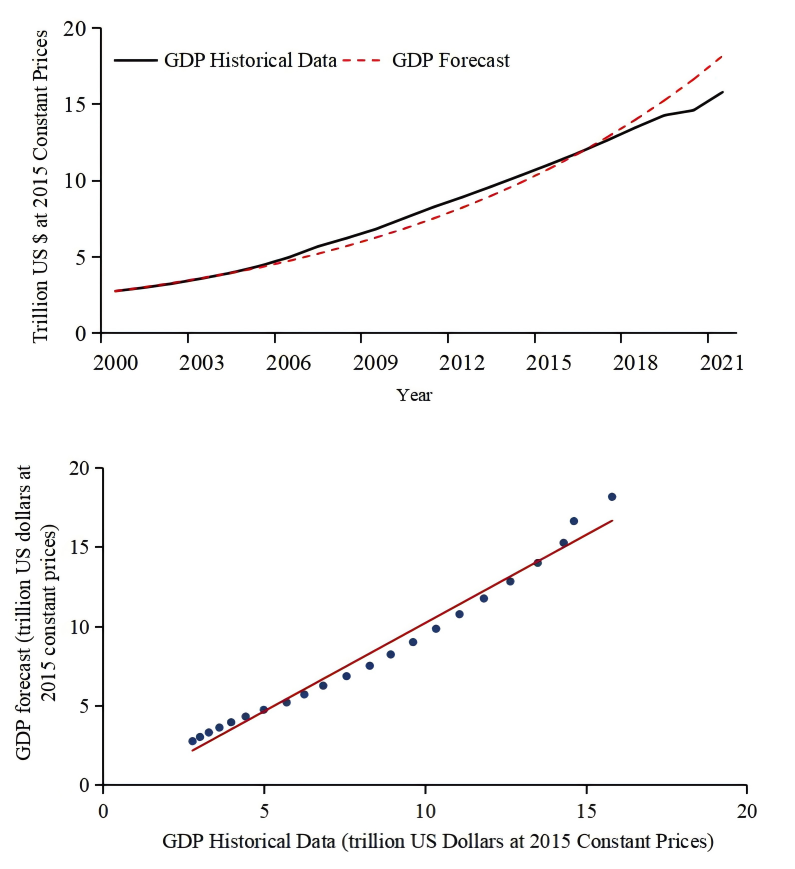


**Figure. A8** Calibration Results for China’s Total GDP Model

### **Note:** Regression lines are estimated using Ordinary Least Squares (OLS) methodology. The red line represents the linear regression result with a slope of 1.1119 (p-value<0.001) .

### Our Total GDP Model’s main advantage lies in its simplicity and efficiency. The calibration results of the model demonstrate an average alignment of 94.71% with historical data (from 2000 to 2021). The linear regression equation is y = 1.1119x - 9E+11, with a high R² of 0.9797.


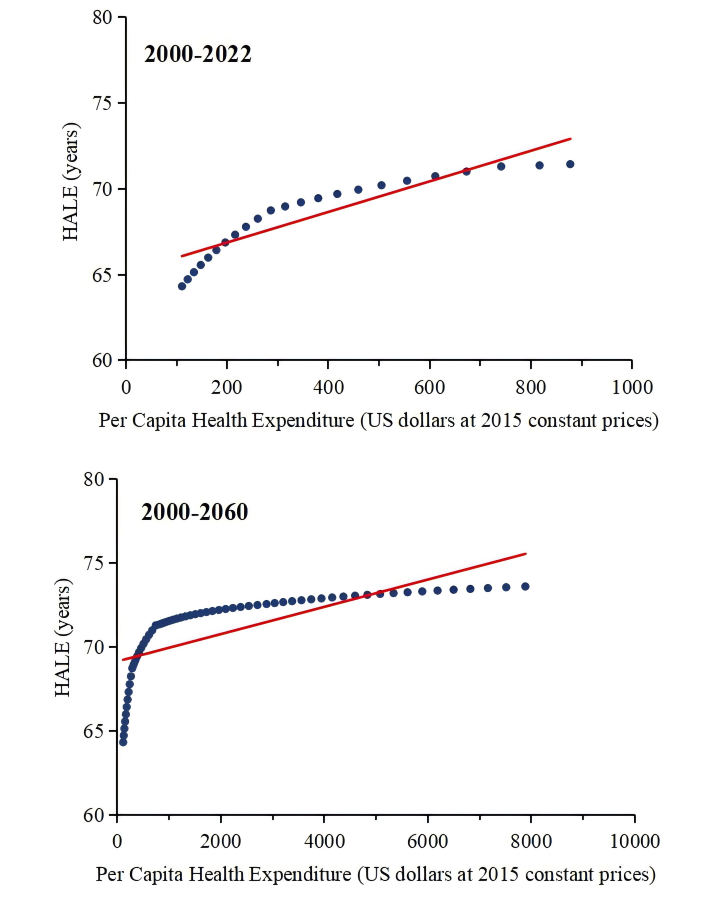


**Figure. A9** Relationship between China’s Per Capita Health Expenditure and HALE at Birth

**Data sources:**

Zhang J, Heffernan M. Using System Dynamics modeling to forecast China’s population until 2060 to visualize the ageing and shrinking population trends. 41st International System Dynamics Conference (ISDC); 2023 Jul 24-28; Chicago, US: System Dynamics Society. Available from: [https://www.iseesystems.com/resources/download/scholarship/2023-zhang.pdf](https://www.iseesystems.com/resources/download/scholarship/2023-zhang.pdf)

Zhang J, Heffernan M. Using System Dynamics to predict health trends in China: A close look at health life expectancy. 42nd International System Dynamics Conference (ISDC); 2024 Aug 4-8; Bergen, Norway: System Dynamics Society. Available from: [https://proceedings.systemdynamics.org/2024/papers/O1020.pdf](https://proceedings.systemdynamics.org/2024/papers/O1020.pdf)

###
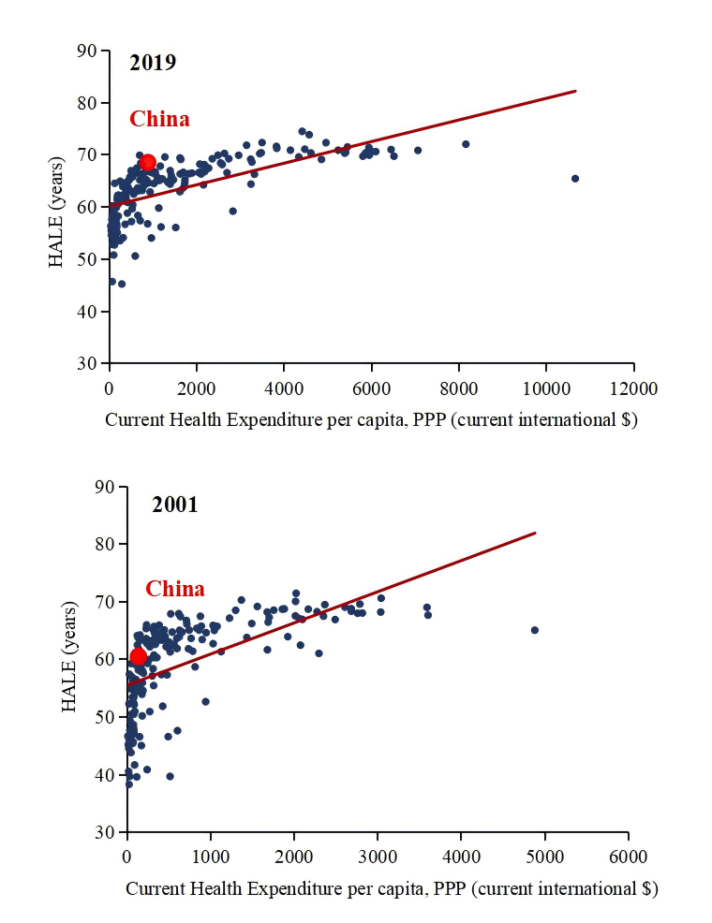


**Figure. A10** Relationship between Per Capita Health Expenditure and Health-Adjusted Life Expectancy at Birth among 185 Global Countries in 2019 and 183 Global Countries in 2001

**Data Sources：**World Bank (2023) and Global Burden Disease (2019)


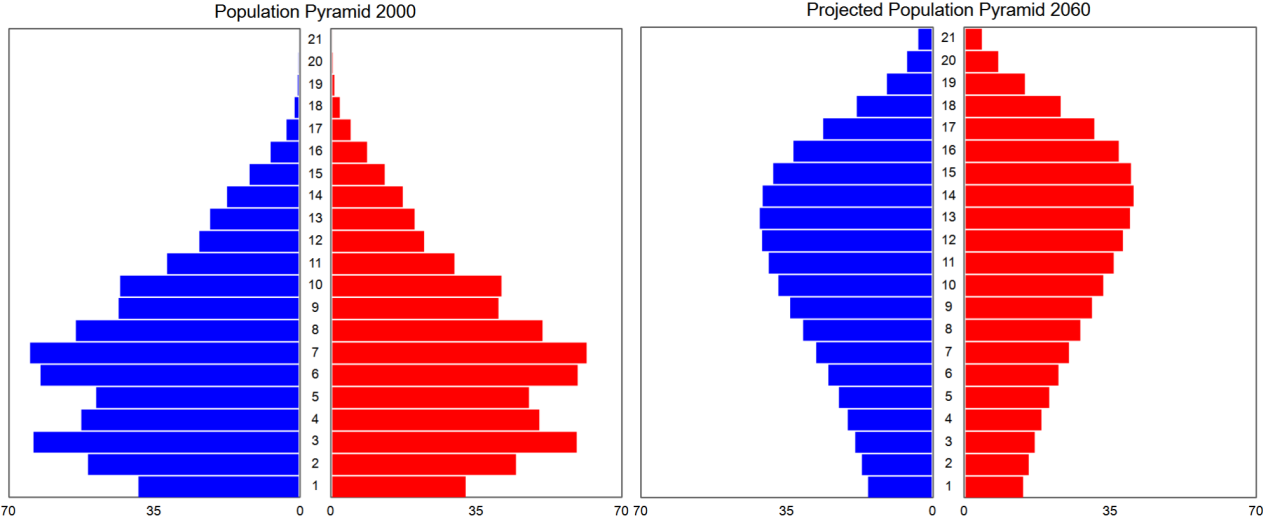


**Figure. A11** Population Pyramid 2000 historical data (left), projected Population Pyramid 2060 using a low TFR scenario (right) [18]

Note: Each unit is one million people. Blue represents males; red represents females. Each bar from 1 to 21 represents a 5-year age group. For example, bar 1 represents newborns to 4.99 years old.

**System Dynamics Simulation Results**
For detailed system dynamics simulation results, please refer to the following link hosted on iseesystems: [https://exchange.iseesystems.com/public/simulation-results/bmc-paper-simulation-results](https://exchange.iseesystems.com/public/simulation-results/bmc-paper-simulation-results/index.html#page1.)

TFR can be adjusted between 1.0 and 3.0. The retirement trend represents three different scenarios for workforce participation rates: 0 (normal), 1 (delayed retirement), and 2 (maximum delayed retirement). The efficiency adjustment factor is set between 0% and 5% per year, and GDP productivity increase can be adjusted from 1% to 9% per year. The projected THE in the paper for 2060 is 8.55 trillion USD, while the simulation result in the link is 8.45 trillion USD. The minor difference from the linked data is due to birth rate settings—our paper uses annual rates, while the linked model uses five-year rates. This difference does not affect the overall conclusions and applies similarly to other indicators with slight differences.
